# Iron starvation induced by vacidobactin A impairs the growth of pyoverdine-deficient *Pseudomonas aeruginosa*

**DOI:** 10.1128/spectrum.01240-26

**Published:** 2026-08-26

**Authors:** Manpreet Kaur, Derek C. K. Chan, Hodan Wardere, Wenliang Wang, Manoj Jangra, Allison K. Guitor, Kalinka K. Koteva, Lori L. Burrows, Gerard D. Wright

**Affiliations:** 1 David Braley Centre for Antibiotics Discovery, McMaster University 3710 , Hamilton, Ontario, Canada; 2 M.G. DeGroote Institute for Infectious Disease Research, McMaster University 3710 , Hamilton, Ontario, Canada; 3 Department of Biochemistry and Biomedical Sciences, McMaster University 3710 , Hamilton, Ontario, Canada; University of Guelph College of Biological Science, Guelph, Ontario, Canada

**Keywords:** *Pseudomonas aeruginosa*, siderophores, vacidobactin, iron acquisition, multidrug resistance, thiostrepton, antimicrobial synergy

## Abstract

Iron acquisition is a crucial step for bacterial survival that shapes bacterial fitness and ecological interactions within microbial communities. *Pseudomonas aeruginosa*, an opportunistic clinical and environmental bacterium, relies on high-affinity siderophores such as pyoverdines to scavenge iron. Understanding these pathways provides vital insights into siderophore specificity, bacterial resource competition, and iron metabolism in bacteria. Here, we report vacidobactin A (VacA), a siderophore produced by the soil bacterium *Variovorax paradoxus*, identified through a screen of natural product extracts targeting a clinical multi-drug-resistant strain of *P. aeruginosa*. Rather than functioning as a direct antimicrobial agent, VacA impairs the growth of P. *aeruginosa* by imposing iron starvation. This is observed exclusively in strains that are unable to produce pyoverdine, their native siderophore. To determine whether VacA-mediated inhibition resulted from restricted access to iron, we heterologously expressed a TonB-dependent transporter from *V. paradoxus* in a pyoverdine- and pyochelin-deficient *P. aeruginosa* strain. Transporter expression enabled utilization of VacA-bound iron and restored growth, demonstrating that the antagonistic effect of VacA is governed by selective siderophore recognition and uptake rather than iron sequestration alone. Additionally, VacA synergized with thiostrepton, which hijacks pyoverdine receptors to enter the cell and inhibit protein synthesis. This investigation demonstrates how environmental microorganisms exploit siderophore specificity to compete for iron and establish antagonistic relationships within microbial communities.

**IMPORTANCE:**

Iron acquisition is fundamental to bacterial survival and shapes ecological interactions within microbial communities, yet the mechanistic principles governing siderophore specificity and iron competition remain incompletely understood. Here, we demonstrate that vacidobactin A, a siderophore produced by *Variovorax paradoxus*, suppresses *P. aeruginosa* growth by limiting iron availability, particularly in strains deficient in pyoverdine production. By demonstrating that heterologous expression of a TonB-dependent transporter restores iron utilization from vacidobactin A, we establish that siderophore-mediated antagonism is governed by selective recognition and uptake pathways. Furthermore, the synergy between vacidobactin A and thiostrepton illustrates how siderophore systems integrate with other cellular vulnerabilities. These findings illuminate fundamental principles of bacterial resource competition and highlight how environmental microorganisms exploit iron metabolism as a competitive strategy, advancing our understanding of microbial community dynamics and bacterial physiology.

## INTRODUCTION

*Pseudomonas aeruginosa* is a remarkably versatile gram-negative bacterium that thrives in diverse ecological niches, ranging from soil and aquatic ecosystems to human tissues, where it acts as a formidable opportunistic pathogen (1, 2). Its intrinsic antibiotic resistance mechanisms, including a poorly permeable outer membrane, numerous broad-specificity efflux pumps, antibiotic-degrading enzymes, the ability to acquire resistance genes through horizontal gene transfer, and its capacity to form drug-insensitive biofilms, collude to limit effective antibiotic treatment (3, 4).

Despite the increasing prevalence of drug-resistant *P. aeruginosa*, the antimicrobial pipeline targeting this pathogen remains critically underdeveloped. Few of the antimicrobial agents in clinical or preclinical development described in a recent World Health Organization report show anti-pseudomonal activity (5), and murepavadin, a once-promising *Pseudomonas-*specific antibiotic, was discontinued in phase III clinical trials due to nephrotoxicity (6). Consequently, there is a need to discover new anti-pseudomonal strategies.

Microbially produced natural products offer a valuable source of antimicrobial chemical matter. Indeed, most antibiotics in clinical use are microbial natural products or their semi-synthetic derivatives. Environmental microbes, in particular, are well-known prolific producers of natural products. We have assembled a collection of thousands of environmental microbes and shown that this is a rich source of antibiotics and other bioactive compounds (7). Because *P. aeruginosa* naturally inhabits complex, competitive environments, it is under constant selective pressure from other soil and aquatic microorganisms. These competitors produce a vast arsenal of bioactive natural products, including siderophores, designed to sequester essential resources such as iron or to suppress competitor growth through metabolic antagonism (1, 8). A screen of a portion of our collection against a clinical strain of multi-drug-resistant (MDR) *P. aeruginosa* identified a vacidobactin family siderophore. We show that vacidobactin A (VacA) suppresses *P. aeruginosa* proliferation by profoundly disrupting its iron homeostasis.

This investigation characterizes iron sequestration as a competitive antagonism mechanism and demonstrates that vacidobactin-induced iron limitation selectively inhibits pyoverdine-deficient *P. aeruginosa*. Interestingly, in pyoverdine-producing strains, VacA still triggers an iron starvation response that increases expression of a TonB-dependent siderophore transporter. This transporter is hijacked by the thiopeptide antibiotic thiostrepton, and its increased expression enhances thiostrepton uptake, resulting in strong synergistic antibacterial activity with VacA. These findings illuminate how metabolic resource limitation drives competitive interactions and creates exploitable physiological vulnerabilities in microbial communities.

## RESULTS

### Identification of VacA from an environmental bacterium

We screened a portion of our collection of natural product-producing microbes (7), focusing on strains derived from a Hamilton, Ontario soil sample that was the source of a previously identified novel lasso peptide antibiotic, lariocidin (9), against *P. aeruginosa* C0292, an MDR strain isolated from sputum. Of >300 methanolic extracts of environmental bacteria isolated from this source, one, MST127, was active against *P. aeruginosa* C0292 (Table S1). MST127 was identified as a strain of the betaproteobacterium *Variovorax paradoxus* based on 16S rRNA and whole-genome sequencing analysis. Bioactivity-guided purification of the fermentation broth yielded the active compound with an *m/z* of 752.3320 [M + H]^+^ determined by high-resolution mass spectrometry, consistent with the molecular formula C_30_H_49_N_7_O_15_ (calculated monoisotopic mass: 752.3309; Fig. 1a). The Chrome Azurol S (CAS) assay suggested that it functions as a putative iron chelator (Fig. S2c).

**Fig 1:**
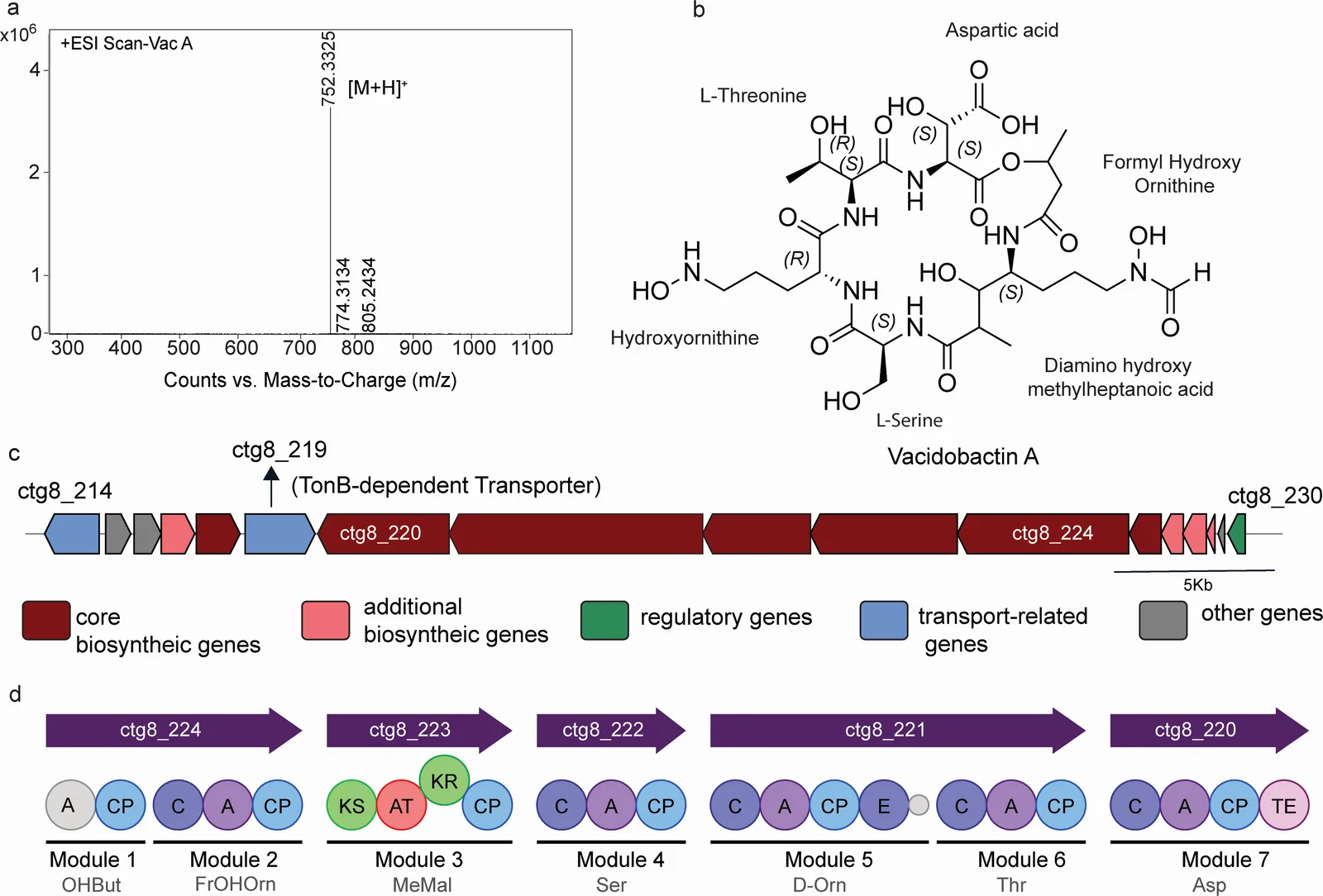
Characterization of VacA. (**a**) High-resolution electrospray ionization mass spectrum (HR-ESI-MS) of purified vacidobactin A (VacA), confirming the expected molecular mass. (**b**) Chemical structure of VacA, annotated to reflect the corresponding amino acid residues. (**c**) Biosynthetic gene cluster of vacidobactin identified in the whole-genome sequence of *Variovorax paradoxus*, as predicted by antiSMASH. Genes are color-coded by function. (**d**) Domain architecture and predicted amino acid specificity of nonribosomal peptide synthetase (NRPS) modules encoded within the vacidobactin BGC.

The structure of the active compound was determined using one- and two-dimensional nuclear magnetic resonance (NMR) spectroscopy coupled with high-resolution electrospray ionization tandem mass spectrometry (HR-ESI-MS/MS). The compound is a cyclic depsipeptide consisting of five amino acids and 3-hydroxybutyric acid (Fig. 1b). The latter is linked via an amide bond to an unusual N-terminal 4-amino-3-hydroxy-7-(*N*-hydroxyformamido)−2-methylheptanoic acid. 3-Hydroxybutyric acid provides the alcohol for intramolecular cyclization via an ester bond with the C-terminal β-hydroxyaspartate (β-OHAsp). The presence of the rare 4-amino-3-hydroxy-7-(*N*-hydroxyformamido)−2-methylheptanoic acid residue in the N-terminus suggested similarities to variochelin (10), imaqobactin (11), variobactin (12), and vacidobactin (12) siderophores from *Variovorax* sp. that incorporate modified 4,7-diamino-3-hydroxy-2-methylpetanoic acid elements. Our structural analysis identified the active compound as a member of the vacidobactin family, a group of β-hydroxyaspartate-acylated siderophores (12). The β-OHAsp and the N-hydroxyornithine provide the key chelating groups necessary for binding Fe (III) ions (13).

The production of vacidobactin A and vacidobactin B has been previously reported from *V. paradoxus* S110 (10). However, their inhibitory activity against *P. aeruginosa* has not been documented. In our initial analyses, the siderophore isolated from the MST127 strain appeared to differ from the published structure of vacidobactin A in the relative positions of Ser and Thr residues, leading us to tentatively designate it as a new variant. To resolve this discrepancy, we obtained the *V. paradoxus* DSM 30334 strain from the original authors and re-isolated vacidobactin A. Detailed NMR and MS analyses demonstrated that the Ser and Thr positions in vacidobactin A from DSM 30334 are identical to those in the compound produced by MST127. This indicates that the previously reported structure of vacidobactin A was misassigned, and the siderophore we identified in MST127 is, in fact, vacidobactin A with a corrected structure (Fig. S1). A direct comparison of the MS and NMR spectra from the MST127-derived compound and DSM 30334-derived vacidobactin A is provided in Fig. S2 to S6. Additional details of structural assignments, including full NMR spectral data sets, MS, and MS/MS analysis, are provided in Fig. S7 to S16 and Table S5. The stereochemistry of the proteinogenic amino acids threonine and serine was experimentally confirmed to be L, whereas ornithine is predicted to be in the D-configuration based on the presence of an epimerase domain in its biosynthetic gene cluster (BGC; Fig. S17).

AntiSMASH (14) analysis of the *V. paradoxus* MST127 genome identified a putative BGC containing both polyketide synthase (PKS) and non-ribosomal peptide synthetase (NRPS) components responsible for its production (Fig. 1c and d). Five core NRPS and PKS-encoding genes, ctg_220 to ctg_224, were identified, providing seven modules predicted to incorporate distinct amino acid and polyketide components. The 4-amino-3-hydroxy-7-(*N*-hydroxyformamido)−2-methylheptanoic acid is presumably generated by *in situ* reaction of NRPS and PKS carrier protein-tethered *N*-formyl-hydroxyornithine and methylmalonic acid components (10). Comparative genomic analysis of the vacidobactin BGCs from MST127 and *V. paradoxus* S110 (DSM 30334; Table S6) revealed conserved enzymatic architectures, including seven PKS/NRPS modules, consistent with the production of closely related siderophore scaffolds.

### Antimicrobial activity profile of VacA against selected bacterial strains

VacA exhibited antimicrobial activity against a subset of *P. aeruginosa* strains from our in-house collection of clinical isolates (strains C0063, C0307, C0222, C0157, C0166, C0292, C0090, and C0060) with minimum inhibitory concentrations (MICs) of 4–32 µg/mL in cation-adjusted Mueller-Hinton broth (CAMHB; Fig. 2a). In contrast, other strains of *P. aeruginosa* (including standard strains such as PAO1 and PA14), *Acinetobacter baumannii*, and *Klebsiella pneumoniae* were insensitive to VacA, with MICs > 128 µg/mL. Although time-kill kinetics studies for VacA at 5× MIC against sensitive *P. aeruginosa* C0060 showed around a 2-log_10_ CFU increase in comparison to the pretreatment level, this demonstrates significantly lower growth compared to the untreated culture control (Fig. 2b). In an additional experiment, optical density was recorded over a period of 18 h using different concentrations of VacA against *P. aeruginosa* C0060, and the results clearly demonstrate the growth-impairing effect of VacA (Fig. 3c), consistent with the mode of action of siderophores, whose antimicrobial activity is governed by iron availability (8, 15).

**Fig 2:**
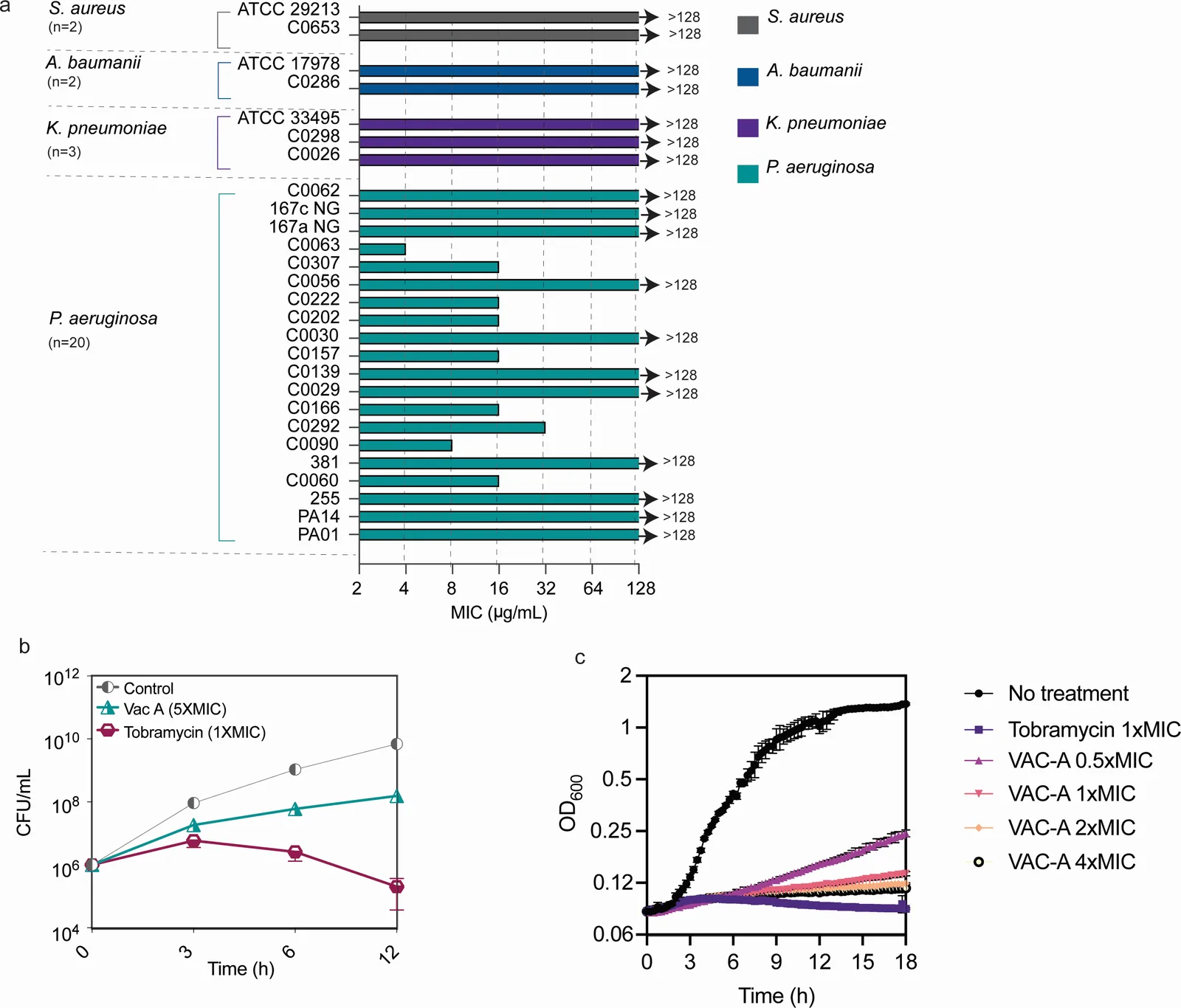
Antimicrobial activity of VacA. (**a**) Minimum inhibitory concentrations (MICs) of VacA against various bacterial strains, including clinical *Pseudomonas aeruginosa* isolates, determined in cation-adjusted Mueller-Hinton broth (CAMHB). (**b**) Time-kill kinetics of VacA against *P. aeruginosa* C0060. Bacterial cultures were treated with VacA, and viable counts were determined at the indicated time points. Data represent means ± standard deviation from three independent biological replicates, each performed in duplicate. (**c**) Time-kill kinetics demonstrating the effect of different concentrations of VacA on the growth of *P. aeruginosa* C0060 based on optical density measurements. Data represent means ± standard deviation from two independent biological replicates, each performed in triplicate.

**Fig 3:**
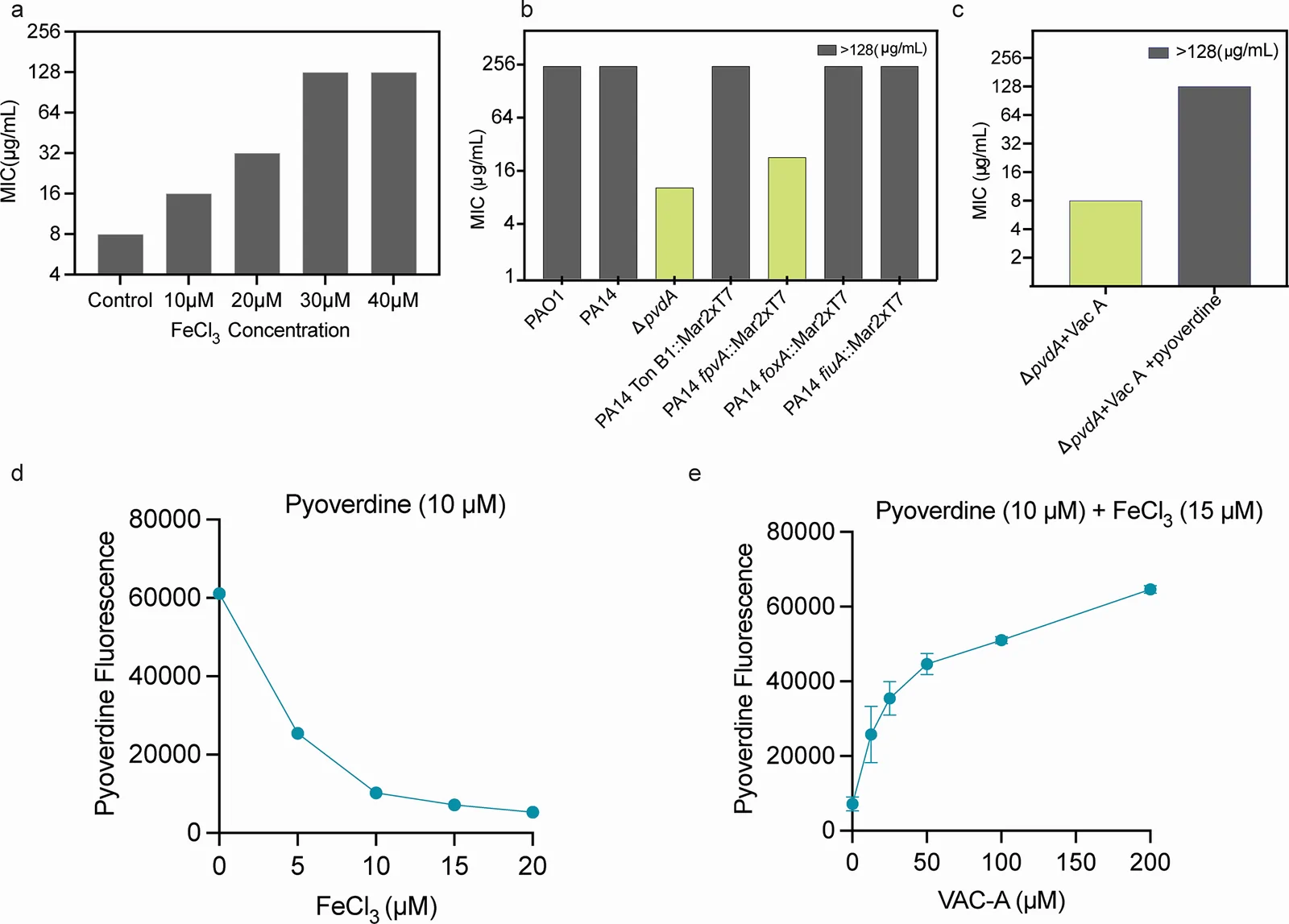
Interaction of VacA with *P. aeruginosa deletion mutant* strains and pyoverdine. (**a**) Minimum inhibitory concentration (MIC) of VacA against *P. aeruginosa* strain C0060 in M9 minimal medium (baseline iron concentration [0.6 µM]) supplemented with increasing concentrations of FeCl₃, compared to control conditions. (**b**) MIC of VacA against *P. aeruginosa* PA14 mutant strains defective in iron uptake pathways. (**c**) Effect of exogenous pyoverdine addition on the MIC of vacidobactin A in the Δ*pvdA* mutant. (**d**) Effect of iron on pyoverdine fluorescence shown as quenching of fluorescence when ferripyoverdine complex is formed. (**e**) Pyoverdine fluorescence competition assay with VacA. Increasing concentration of VacA restores pyoverdine fluorescence by competing with Fe^3+^.

### VacA inhibits *P. aeruginosa* via iron starvation in pyoverdine-deficient strains

The role of iron availability in VacA’s anti-pseudomonal activity was investigated by determining MICs in the presence and absence of supplemental iron (FeCl₃). In iron-limited M9 minimal medium (~0.6 µM Fe³^+^), the MIC for *P. aeruginosa* C0060 was 8 µg/mL. However, the MIC increased to 16 µg/mL and 32 µg/mL with the addition of 10 µM and 20 µM FeCl₃, respectively, and >128 µg/mL with the addition of 30 µM and 40 µM FeCl₃ (Fig. 3a). An increase in the MIC of VacA after adding iron to the media suggested that VacA was interfering with iron uptake or metabolism, thereby inhibiting the growth of sensitive strains of *P. aeruginosa*. To investigate this selective sensitivity further, we compared the MICs of VacA-insensitive *P. aeruginosa* PA14 strains with those of mutants from a PA14 transposon library (15). Transposon insertions in *pvdA,* essential for producing the intrinsic siderophore pyoverdine, and *fpvA*, which is the primary pyoverdine receptor, conferred susceptibility to VacA (Fig. 3b). Supplementation with pyoverdine alone (Fig. 3c) or pyoverdine and VacA in combination restored growth, suggesting that pyoverdine competes with VacA for iron. To demonstrate that pyoverdine has a superior iron affinity compared to VacA, we performed an assay in which pyoverdine’s intrinsic fluorescence is quenched when binding to Fe^3+^ (16); VacA restored pyoverdine fluorescence in a concentration-dependent manner in the presence of ferric iron, indicating that VacA competes with pyoverdine for Fe^3+^ (Fig. 3d). However, near-complete fluorescence recovery required a substantial molar excess of VacA, with 200 µM VacA yielding approximately 90%–95% of the fluorescence observed for pyoverdine in the absence of iron (Fig. 3e). These data suggest that although VacA can sequester ferric iron, it does so less effectively than pyoverdine under the assay conditions, consistent with a lower apparent iron-binding competitiveness. This provides a plausible explanation for why pyoverdine-producing strains are less susceptible to the growth-inhibitory effects of VacA, and why the addition of exogenous pyoverdine can rescue growth in VacA-sensitive strains by outcompeting VacA for available iron.

We conducted another experiment to evaluate pyoverdine production. A panel of strains isolated from various clinical samples was grown under iron-limited conditions, and fluorescence was measured relative to a positive control (PA14) and a negative control (∆*pvdA*). As shown in Fig. S18, most isolates produced substantial pyoverdine, while a subset showed minimal or no fluorescence, indicating impaired or absent pyoverdine biosynthesis. Notably, these strains were sensitive to VacA, as indicated in Fig. 2a.

### Expression of a TonB-dependent transporter of *V. paradoxus* in *P. aeruginosa* PA14 ∆*pvdA* ∆*pchA* enables VacA-dependent iron uptake

The VacA BGC of *V. paradoxus* MST 127 encodes a predicted TonB-dependent transporter that we hypothesized is responsible for the uptake of the VacA-Fe^3+^ complex (Fig. 1c). The TonB-dependent transporter from *V. paradoxus* was expressed in the *P. aeruginosa* PA14 ∆*pvdA* ∆*pchA* mutant to explore whether VacA can be used as an iron source in the absence of native siderophores. This strain lacks the ability to synthesize pyoverdine (due to deletion of pvdA) and pyochelin (due to deletion of pchA), the two primary siderophores produced by *P. aeruginosa*, rendering it unable to acquire iron via its endogenous siderophore pathways. The ferrichrome transporter, FiuA, from *P. aeruginosa* PA14 most closely resembles the putative *V. paradoxus* VacA transporter and shares 40% identity (Fig. 4a). In addition, ferrichrome and vacidobactin share partial structural features characteristic of hydroxamate-type siderophores, providing a rationale for using FiuA as a control transporter in this study. The predicted vacidobactin A transporter could not be functionally expressed in the siderophore-deficient mutant because the N-terminus is important for TonB recognition. Therefore, we generated a chimera that replaced the N-terminus of the *V. paradoxus* TonB-dependent transporter with that of FiuA (PA14_06160) to ensure functionality in *P. aeruginosa* (Fig. 4b). The control strains *P. aeruginosa* PA14 ∆*pvdA* ∆*pchA* pHERD20T (empty vector) and *P. aeruginosa* PA14 ∆*pvdA* ∆*pchA* pHERD20T-*fiuA* were sensitive to VacA with MIC values of 16 µg/mL, while PA14 ∆*pvdA* ∆*pchA* pHERD20T-N*_fiuA_-*MST 127 was insensitive to VacA with a MIC > 128 µg/mL (Fig. 4c).

**Fig 4:**
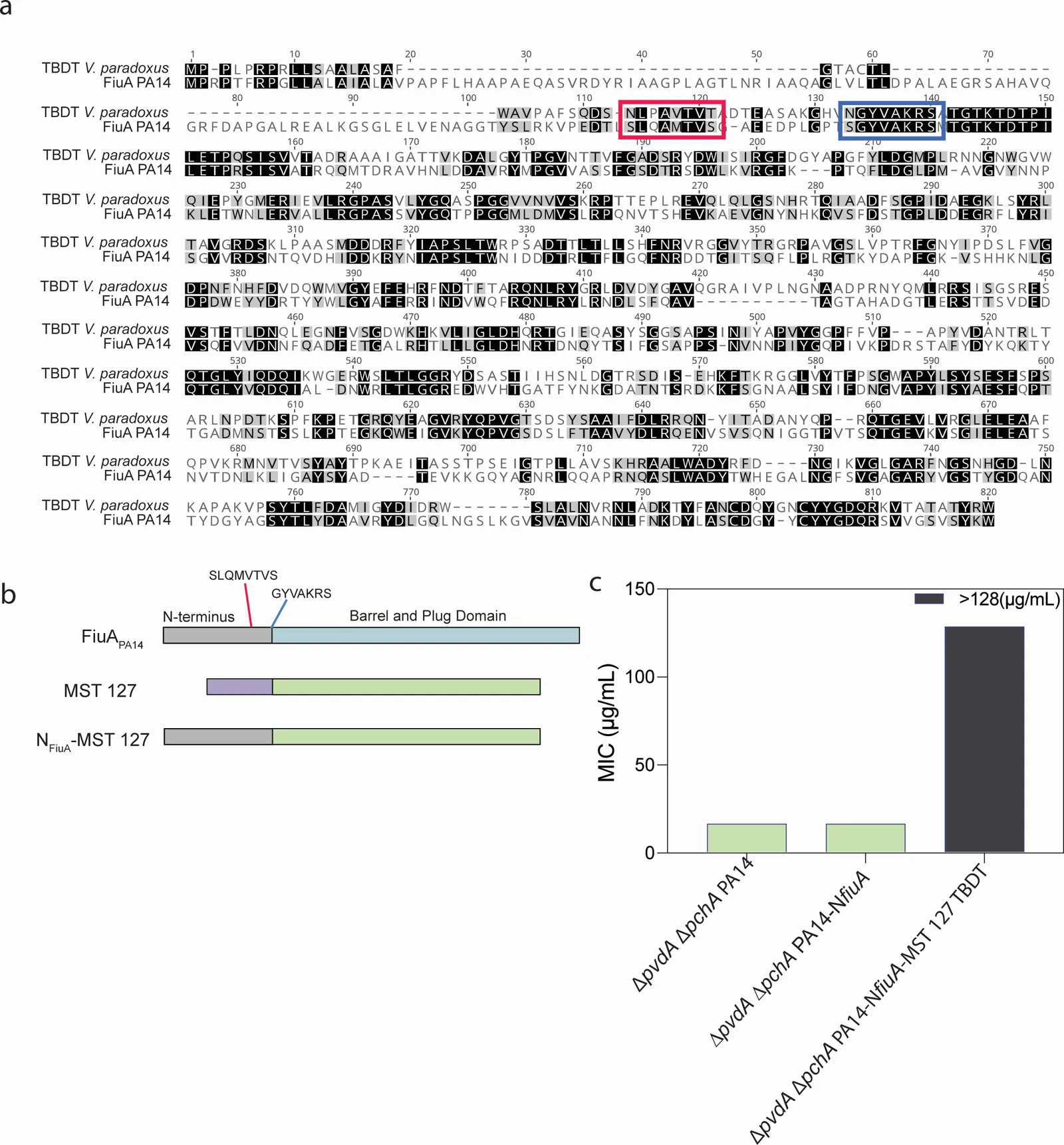
Genetic and functional characterization of VacA biosynthesis and TonB-dependent transport. (**a**) The sequence alignment of the FiuA TonB-dependent transporter from *P. aeruginosa* PA14 (PA14_06160) and the TonB-dependent transporter from *Variovorax paradoxus* shows 40% identity. The TonB box is highlighted in red, and the overlap region is highlighted in blue. (**b**) Graphical representation of the fusion of the two transporter sequences, with the TonB box of PA14 FiuA indicated in red and the overlap region in blue. (**c**) MIC of mutant strains expressing the ∆*pvdA* ∆*pchA* pHERD20T (empty vector), ∆*pvdA* ∆*pchA* pHERD20T--N*_fiuA_-*, and ∆*pvdA* ∆*pchA* pHERD20T-N*_fiuA_-*MST 127 TBDT (chimeric transporter N-FiuA-MST 127). The producer strain demonstrates the receptor’s functional role in VacA activity.

These results are consistent with the chimeric transporter facilitating VacA-dependent iron uptake in a manner that supports growth under the conditions tested.

### VacA potentiates the activity of thiostrepton against *Pseudomonas* strains

Previous studies demonstrated that iron starvation induces production of the pyoverdine receptors FpvA or FpvB, which unexpectedly facilitated the uptake of large, otherwise impermeant antibiotics such as thiostrepton (17). To evaluate the potential of VacA as an antibiotic adjuvant, we performed synergy assays to measure the combined effect of thiostrepton and VacA against *P. aeruginosa*, including standard strains (PAO1 and PA14) and clinical isolates, including pyoverdine producers (C0062 and C0030) and non-producers (C0060 and C0090). Synergistic interactions were observed for all tested strains, with pronounced synergy in pyoverdine-producing isolates and reduced synergy in non-producers, likely reflecting their individual MICs (Fig. 5a through f).

**Fig 5:**
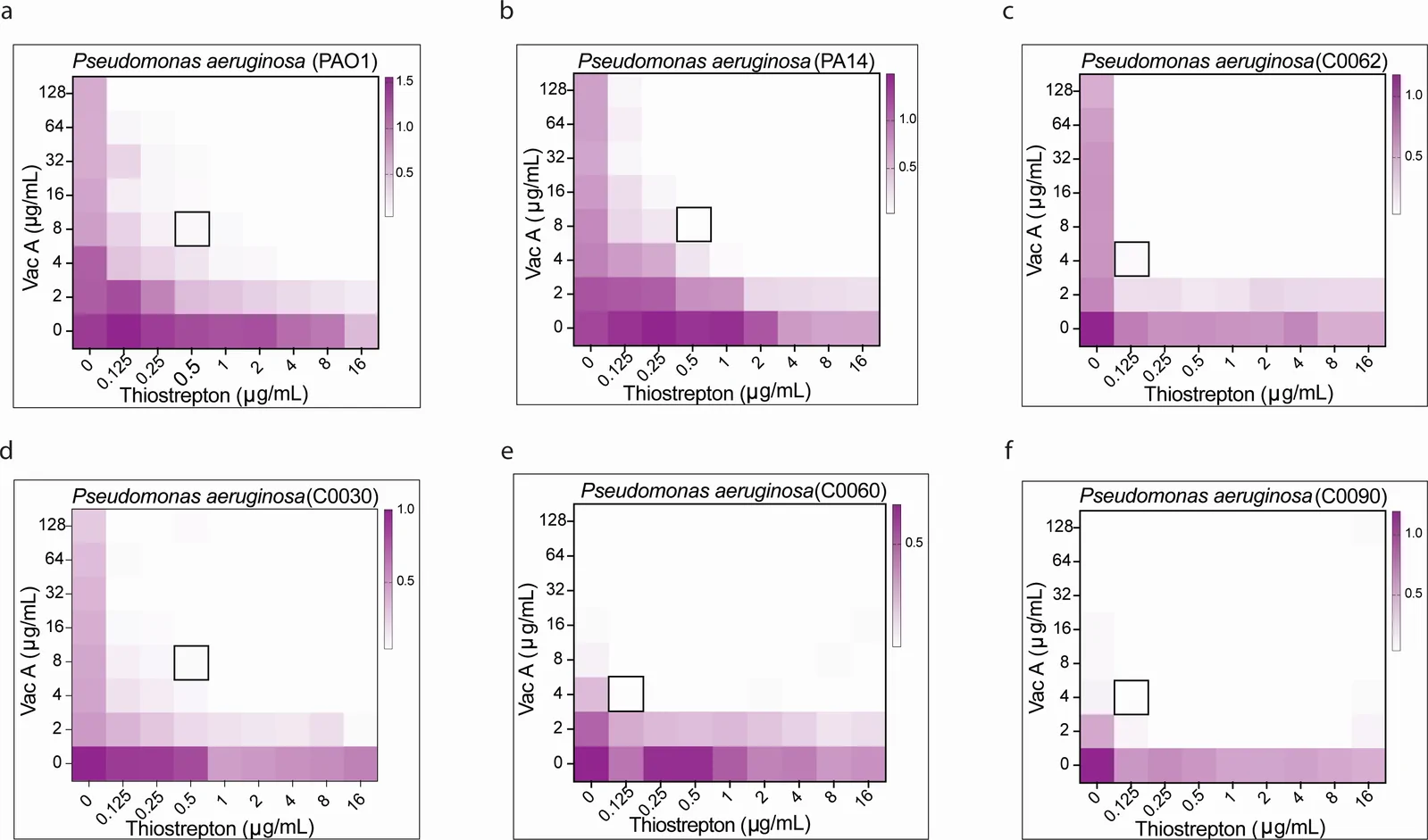
Synergistic antibacterial activity of VacA and thiostrepton against *P. aeruginosa* strains, including clinical isolates. (**a–f**) Synergy assay heatmaps illustrating the combined effects of thiostrepton and VacA against *P. aeruginosa* strains, including laboratory strains PAO1 and PA14, and clinical isolates C0062 and C0030 (pyoverdine producers), and C0060 and C0090 (non-pyoverdine producers). Concentrations of thiostrepton (*x*-axis) and VacA (*y*-axis) are indicated for each panel. Growth inhibition is represented by shading intensity, with lighter colors indicating greater inhibition. Regions of synergistic interaction, where subinhibitory concentrations of both compounds lead to pronounced growth inhibition, are highlighted with black boxes. The data shown represent one of three independent biological replicates, each yielding results comparable to the others. We assigned approximate MIC values of 256 µg/mL for VacA and 32 µg/mL for thiostrepton based on the highest tested concentrations. FICI values (VacA/thiostrepton combinations) were as follows: PAO1 (0.0469), PA14 (0.0469), C0062 (0.0195), C0030 (0.0469), C0060 (0.2539), and C0090 (0.5039).

The combination of VacA (8 µg/mL) and thiostrepton (0.5 µg/mL) significantly suppressed growth of a panel of 21 clinical *P. aeruginosa* isolates. As shown in Fig. S19, the extent of inhibition varied among isolates, but overall, the combination consistently reduced bacterial growth. These findings highlight the efficacy of VacA and thiostrepton as a potential antimicrobial combination, particularly against pyoverdine-producing *P. aeruginosa* strains.

### VacA enhances pyoverdine production and increases receptor availability for thiostrepton uptake

To assess the effect of VacA on pyoverdine production, we treated *P. aeruginosa* PA14 with VacA (8 µg/mL), thiostrepton (0.5 µg/mL), or their combination. Optical density measurements (OD₆₀₀) revealed that PA14 grew well under control conditions and showed minimal growth inhibition with thiostrepton alone. However, VacA treatment significantly reduced growth, with OD₆₀₀ values approximately half of the control (Fig. 6a).

**Fig 6:**
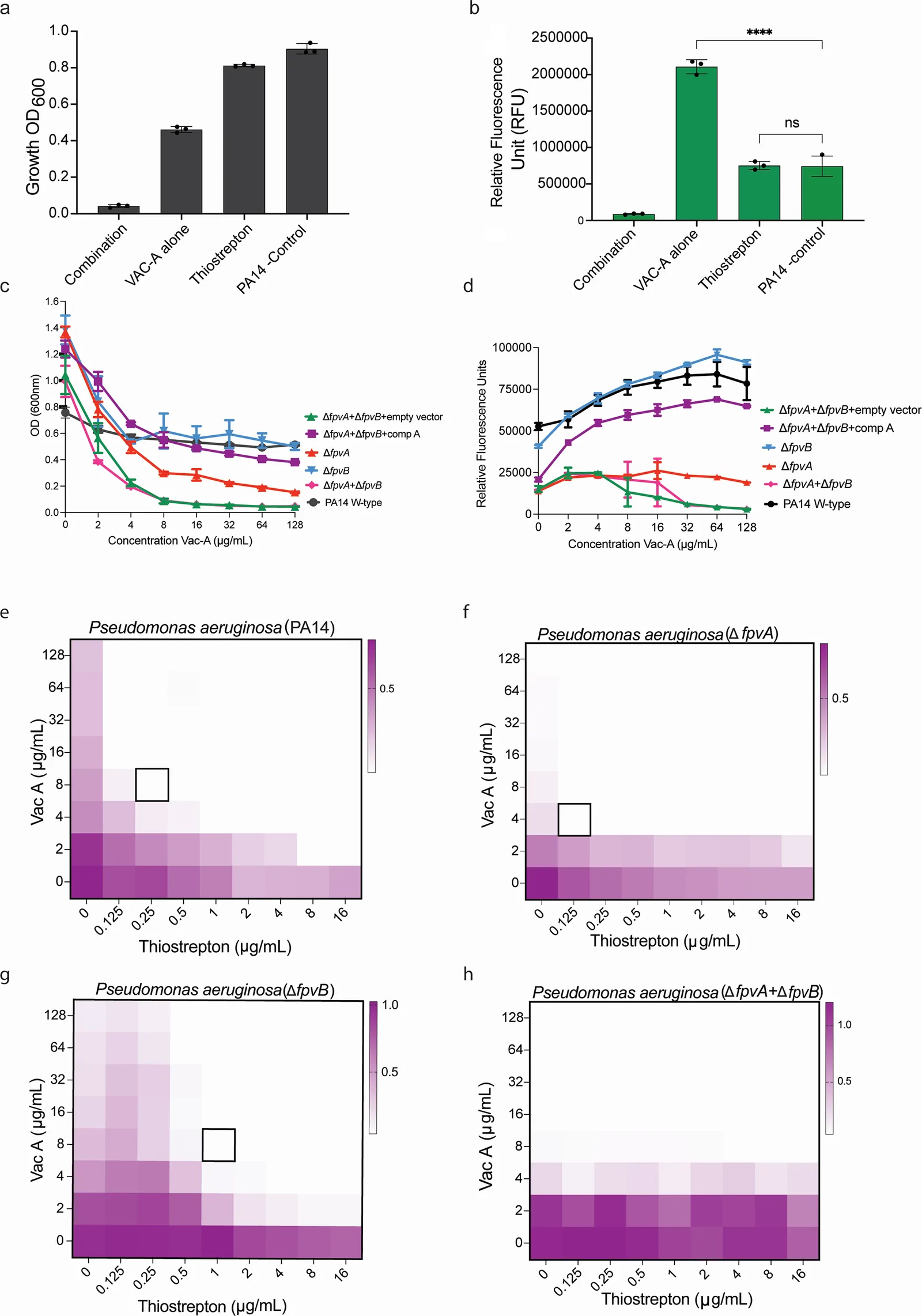
VacA enhances pyoverdine production and promotes thiostrepton uptake via siderophore receptors. (**a**) Growth (OD₆₀₀) of *P. aeruginosa* PA14 after 18 h treatment with VacA (8 µg/mL), thiostrepton (0.5 µg/mL), or a combination of the two. (**b**) Pyoverdine fluorescence following treatment, showing increased pyoverdine production in response to VacA alone. (**c**) Growth inhibition assay showing optical density (OD₆₀₀) measurements of *P. aeruginosa* strains exposed to increasing concentrations of VacA (0–128 µg/mL). Wild-type PA14, single siderophore receptor mutants (Δ*fpvA* and Δ*fpvB*), the double mutant (Δ*fpvA*Δ*fpvB*), and the double mutant carrying either an empty vector or complementation construct (Δ*fpvA*Δ*fpvB* + comp A) were assessed. Complementation with the transporter restored VacA-mediated growth inhibition compared with the empty vector control, supporting transporter-dependent uptake and strain-specific sensitivity to iron sequestration by VacA. (**d**) Pyoverdine-associated fluorescence (RFU) was quantified in *P. aeruginosa* PA14 and receptor mutant strains exposed to increasing concentrations of VacA (0–128 µg/mL). (**e–h**) Synergy between VacA and thiostrepton in the deletion mutant strains. Data represent the mean ± SD of at least three independent experiments. *P* values were determined using Dunnett’s multiple comparisons test; *P* < 0.0001 for (**b**) FICI values (VacA/thiostrepton combinations) were as follows: PA14 (0.039), ΔfpvA (0.2539), and ΔfpvB (0.0625).

Next, we examined pyoverdine production under the same conditions. VacA alone markedly induced pyoverdine synthesis, whereas thiostrepton had no effect (Fig. 6b). These results suggest that VacA enhances pyoverdine production, potentially increasing the availability of siderophore receptors needed for thiostrepton uptake. In *P. aeruginosa*, ferripyoverdine binding to the outer membrane receptor FpvA activates the extracytoplasmic function sigma factor PvdS, which coordinately upregulates both pyoverdine biosynthesis genes and *fpvA* expression, thereby establishing a positive feed-forward regulatory loop (18). Increased pyoverdine production would therefore be expected to increase FpvA receptor abundance, potentially enhancing thiostrepton uptake.

To investigate whether sensitivity to VacA depends on iron uptake specificity, growth responses of *P. aeruginosa* PA14, siderophore receptor mutants, and complemented strains were evaluated across increasing concentrations of VacA. While the double knockout strain Δ*fpvA*Δ*fpvB* showed almost complete inhibition, single-gene knockout strains for either receptor showed differential susceptibility profiles. Δ*fpvA* had a profound impact on growth in the presence of VacA, whereas Δ*fpvB* behaved like parent PA14. Expression of the receptor FpvA back in the Δ*fpvA*Δ*fpvB* background restored sensitivity compared with the empty vector control (Fig. 6c). FpvA is the primary receptor for pyoverdine-mediated iron uptake in *P. aeruginosa*; in the *fpvA* mutant, pyoverdine cannot be efficiently re-imported as a pyoverdine–Fe³^+^ complex, rendering it non-functional for iron acquisition. Nevertheless, pyoverdine can still be imported through the less-efficient alternative receptor FpvB. However, this strain showed a significant growth defect in the presence of VacA at concentrations where it inhibits the growth of pyoverdine-non-producing strains. This observation led us to test pyoverdine production in this strain. Indeed, the *fpvA* deletion mutant showed a reduced level of pyoverdine compared to wild-type PA14 (Fig. 6d), consistent with previous reports (19), suggesting that low levels of pyoverdine in combination with a less-efficient uptake system result in VacA sensitivity. Interestingly, both Δ*fpvA* and Δ*fpvB* mutants had increased levels of pyoverdine in the presence of VacA in a concentration-dependent manner compared to untreated cultures (Fig. 6d), indicating that they should now be sensitive to thiostrepton due to overexpression of the corresponding receptor.

To test this hypothesis, we evaluated the synergy between VacA and thiostrepton in PA14 mutant strains lacking either the pyoverdine receptors FpvA or FpvB (Fig. 6e through h). As expected, both strains showed comparable susceptibility to the combination as parent PA14. In contrast, no synergy was obtained in the double knockout strain, supporting the importance of these receptors for thiostrepton uptake. However, we noticed that a two- to fourfold higher concentration of thiostrepton is required to inhibit the ∆*fpvB* mutant in the presence of a similar concentration of VacA, compared to the ∆*fpvA* mutant, suggesting thiostrepton prefers uptake by the FpvB receptor.

## DISCUSSION

This study demonstrates that VacA, a siderophore produced by environmental *V. paradoxus*, impairs growth of the pyoverdine-deficient *P. aeruginosa* strains through iron starvation. Furthermore, VacA shows a potent synergistic effect with the large-scaffold antibiotic thiostrepton across a broad range of *P. aeruginosa* isolates, regardless of their pyoverdine production status. VacA was originally isolated and characterized by Johnson et al. (12) from *V. paradoxus* S110 strain; however, no antimicrobial activity was reported. This may be due to the absence of VacA activity against standard *P. aeruginosa* strains, such as PAO1 and PA14, which are often used for antibacterial screening. We identified VacA during a targeted screen of a multidrug-resistant *P. aeruginosa* clinical isolate, C0292, which does not produce pyoverdine. Pyoverdine deficiency is not uncommon in clinical settings, particularly among isolates from chronic infections. Studies have reported that pyoverdine-deficient *P. aeruginosa* isolates emerge during long-term CF lung infections, with some studies documenting that ~30% of CF isolates have reduced or absent pyoverdine production (20, 21). These data highlight the impact of genetic variability among clinical isolates, including collateral sensitivity, in which resistance to one antibiotic may confer hypersensitivity to an unrelated compound (22). Our results also emphasize the critical role of indicator strain selection in antimicrobial discovery efforts and support the idea that screening against a broader and more phenotypically diverse strain set can uncover activities that would be missed with standard laboratory reference strains alone. Our structural re-characterization of VacA corrects the previously misassigned positions of serine and threonine residues in the originally reported structure. This correction, confirmed through comparative analysis with the reference strain DSM 30334, highlights the importance of rigorous structural validation in natural product research.

Iron acquisition is a central determinant of *P. aeruginosa* fitness in both environmental and host-associated settings, including in biofilm formation, where iron is frequently limiting and tightly sequestered (23–25). To survive under iron-limiting conditions, *P. aeruginosa* produces the high-affinity iron-chelating compounds pyoverdine and pyochelin. The selective activity of VacA against pyoverdine-deficient *P. aeruginosa* strains reveals an important vulnerability in this notoriously drug-resistant pathogen. Pyoverdine exhibits exceptionally high ferric iron affinity (Ka ≈ 10³² M⁻¹), far exceeding VacA, whereas pyochelin (Ka ≈ 10⁵ M⁻¹) (26, 27) is too weak to compete effectively. We therefore employed a ∆*pvdA* ∆*pchA* double knockout to eliminate residual siderophore activity and unambiguously attribute growth inhibition to VacA-mediated iron sequestration. Heterologous expression of the *V. paradoxus* TonB-dependent transporter in a pyoverdine- and pyochelin-null *P. aeruginosa* background restored growth in the presence of vacidobactin, confirming that VacA activity relies on transporter-specific iron uptake rather than rendering iron universally inaccessible. Our findings demonstrate that VacA’s inhibitory activity results from competitive iron chelation, effectively starving bacteria that cannot produce their primary siderophore, pyoverdine. This iron-dependent activity is evidenced by the dose-dependent reversal of growth inhibition upon iron supplementation and the restoration of growth when pyoverdine is provided exogenously. The selective nature of VacA’s activity aligns with the ecological context in which it is produced. *V. paradoxus*, commonly found in soil and aquatic environments where *P. aeruginosa* also thrives, may have evolved VacA production to provide a competitive advantage for iron acquisition in these shared habitats. These results align with previous reports demonstrating the antimicrobial activity of siderophores, such as variochelins, produced by various *Variovorax* species (28). While previous studies did not specifically test variochelins against *P. aeruginosa*, the demonstrated antimicrobial activity against other gram-negative bacteria aligns with our findings and suggests that *Variovorax*-derived siderophores may represent a broader class of competitive iron-chelating antimicrobials. The structural similarities between variochelins and VacA, particularly the presence of β-hydroxyaspartate and N-hydroxyornithine chelating groups, likely contribute to their shared ability to disrupt bacterial iron homeostasis.

Siderophores produced by *P. aeruginosa* require specific TonB-dependent transporters to cross the outer membrane and access the periplasm and cytoplasmic membrane for further transport into the cell (29, 30). Several antimicrobial natural products, including siderophore-antibiotic conjugates such as cefiderocol, can act as Trojan horses, exploiting these iron transporters to enter cells. For instance, the bulky hydrophobic thiopeptides thiocillin and micrococcin hijack the ferrioxamine receptor FoxA (31), while thiostrepton exploits the pyoverdine receptors FpvA and FpvB(17). Iron chelators potentiate these antimicrobial compounds by inducing iron-limiting conditions, thereby increasing the expression of relevant siderophore receptors. This phenomenon underlies the efficacy of VacA in starving iron availability in combination with the antibiotic thiostrepton and suggests potential synergy with other siderophore-receptor-dependent antibiotics, such as the U.S. Food and Drug Administration-approved cefiderocol. Similar results can be expected with other siderophores as well, such as ethylenediaminedi(o-hydroxyphenylacetic acid) (EDDHA), which is a well-characterized synthetic iron chelator with comparable properties to VacA. Critically, pyoverdine-deficient strains of *P. aeruginosa* are unable to grow in iron-deficient minimal medium containing EDDHA, demonstrating the chelator’s efficacy at blocking iron acquisition (32). However, supplementation with alternative siderophores (such as enterobactin) can restore growth, indicating that pyoverdine is the primary mechanism for EDDHA-bound iron utilization in this organism (32).

Importantly, VacA demonstrated stronger synergistic activity with thiostrepton than previous studies that explored other siderophores in combination (17, 31, 33). Moreover, the synergistic effect was retained in nutrient-rich conditions (Mueller-Hinton Broth), whereas other studies utilized iron-limiting conditions for the combination. Our data robustly support this established model, which uses thiostrepton or other antibiotics that utilize this uptake mechanism in combination with iron chelators. Critically, we demonstrate that antagonism depends fundamentally on the target strain’s iron-acquisition repertoire—VacA potently inhibits *P. aeruginosa* lacking pyoverdine production by creating severe iron-limiting conditions, while in wild-type strains, it triggers a robust iron starvation response. Importantly, VacA-induced iron limitation synergizes with the translation inhibitor thiostrepton, suggesting that iron-starved cells possess altered physiological vulnerabilities. By elucidating how siderophore-mediated antagonism disrupts iron homeostasis and triggers context-dependent physiological remodeling, this work advances our understanding of competitive interactions in complex microbial communities and reveals how metabolic stress responses can create exploitable cellular vulnerabilities.

## MATERIALS AND METHODS

### Bacterial strains and culture conditions used in this study

The strains, plasmids, and oligonucleotide primers used in this study are listed in Tables S2 to S4. *V. paradoxus* strain MST 127 was grown in ISP2 medium (yeast extract [Difco] 4.0 g/L, malt extract [Difco] 10.0 g/L, dextrose [Difco] 4.0 g/L, pH 5.6) with 1% glycerol and in a shaking incubator at 30°C for 48 h. MIC experiments were performed in CAMHB (BD Biosciences), modified M9 medium (6.8 g/L, Na_2_HPO_4_, 3 g/L, KH_2_PO_4_, 0.5 g/L, NaCl, 1 g/L NH_4_Cl, 0.4% glucose, 2 mM MgSO_4_, 0.1 mM CaCl_2_, 0.2% casein amino acids, and 16.5 μg/mL thiamine) and CAA (casamino acid medium, composition: 5 g/L low-iron CAA [Difco], 1.46 g/L K_2_HPO_4_ 3H_2_O, and 0.25 g/L MgSO_4_ 7H_2_O) (34). Lennox Broth (LB; Bioshop) was used to test the intracellular iron level experiments. All strains were incubated at 37°C.

### Screening of soil isolates and activity-guided purification of vacidobactin A

Bacterial strains isolated from soil samples were screened for their antimicrobial activity against the multidrug-resistant clinical strain of *P. aeruginosa* C0292. Tryptic soy broth TSB, half-strength brain heart infusion broth, and minimal media supplemented with peptones were selected for soil isolate fermentation. One milliliter of cell-free broth was collected after 24 h, 48 h, 5 days, and 10 days of incubation, lyophilized overnight, and resuspended in 50% DMSO (50–70 µL). For antimicrobial activity screening, *P. aeruginosa* was inoculated on Mueller-Hinton agar plates, 5 µL of the DMSO extract was applied, and plates were incubated at 37°C overnight. The extract from *V. paradoxus* MST 127 grown for 48 h in minimal media supplemented with peptones showed consistent activity against *P. aeruginosa*. ISP2 medium with 1% glycerol was selected for further purification as it yielded the highest antimicrobial activity. A 50 mL seed culture was used to inoculate 3 L of medium. After 2 days of incubation at 30°C, the supernatant was separated from the cells by centrifugation at 3,400 × *g* for 20 min. The cell-free supernatant was added to 8% (wt/vol) of activated Diaion HP-20 (Sigma) resin and mixed for 2–3 h. The resin was filtered and washed with distilled water. The compounds bound to the resin were eluted with 100% methanol after 2 h of mixing, and the solvent was removed using a rotary evaporator. The extract was reconstituted in Milli-Q water, loaded onto a Sephadex LH-20 column, and eluted with a water-methanol gradient from 0% to 50% methanol. The antibiotic activity of these fractions was assessed using the well-diffusion method. The active fractions were pooled and loaded on a reverse-phase CombiFlash ISCO column (RediSep Gold Rf C18, Teledyne). The compounds were eluted with acetonitrile (ACN) and water using a 0%–95% ACN gradient. The active fractions were dried and then applied to an HPLC C28 Analytical column (Sunniest RP-AquA C28 4.6 × 100 mm, 5 µm) using water and eluted with a gradient of ACN. Both solvents were supplemented with 0.1% trifluoroacetic acid. The fractions were collected and assessed for antimicrobial activity. The active peak eluted at approximately 7% ACN. The pure compound, vacidobactin A, was lyophilized to generate a white powder. A similar purification procedure was followed to isolate VacA from the DSM 30034 strain.

### Structural characterization of VacA

The mass and MS/MS data of VacA were analyzed using HR-ESI-MS recorded with an Infinity II LC System (Agilent Technologies) coupled with a qTOF 6550 mass detector in positive ion mode. For NMR spectroscopy, 8 mg of pure VacA from MST 127 and 5 mg from DSM 30034 were dissolved in deuterated DMSO, and 1D and 2D NMR spectra were acquired on a Bruker AVIII 700 MHz instrument equipped with a cryoprobe.

### CAS assay

CAS agar plates were prepared following a previously established method (35). VacA was adjusted to a final concentration of 2 mg/mL, and 10 µL of each sample was carefully spotted onto the agar surface. Producer strains of iron chelators were used for comparison. The plates were then incubated at room temperature for 1 h, after which the results were recorded.

### Determination of the stereochemistry of VacA amino acids by Marfey analysis

To a solution of VacA (0.6 mg) was added 6 N HCl (1 mL). Peptide bond hydrolysis was carried out for 20 h at 90°C with stirring in a closed glass vessel. The sample was then diluted with water and freeze-dried. To the hydrolyzed dry peptide was added water (10 µL), followed by 1 M sodium bicarbonate (10 µL) and (1-fluoro-2,4-dinitrophenyl-5-L-alanine amide; Marfey’s reagent; 50 µL of 1% solution in acetone). The reaction was carried out for 1 h at 40°C and terminated by the addition of 1 M HCl (10 µL) and methanol (420 µL), followed by LC-MS analysis (36).

### Marfey analysis of reference serine and threonine amino acids

To 10 µL of 10 mg/mL solution of the corresponding amino acid L or DL was added 10 µL of 1 M sodium bicarbonate (10 µL) and Marfey’s reagent (50 µL of 1% solution in acetone). The reaction was carried out as described above. High-resolution electrospray ionization mass spectra were acquired using an Agilent 1290 UPLC separation module and a qTOF 6550 mass detector in positive ion mode. For LC separation, an Agilent Eclipse XDB-C8 column (2.1 × 150 mm; 3.5 µm) and the following method were used: from 0 to 0.5 min, 90% A (0.1 [vol/vol] formic acid in water), 10% B (acetonitrile), from 0.5 to 1 min up to 20% B followed by a linear gradient from 1 to 20 min to 30% B, then from 20 to 25 min sharp increase to 90%B at a flow rate of 0.2 mL/min.

### Biosynthetic gene cluster identification

The genome sequence of *V. paradoxus* MST 127 was obtained by the hybrid assembly of Illumina MiSeq reads and Nanopore data. The VacA biosynthetic gene cluster was identified using antiSMASH 5.0. (14).

### MIC assay

MICs were determined using the Clinical and Laboratory Standards Institute broth microdilution method (37) in a final volume of 100 µL in 96-well U-bottom plates. CAMHB and M9 minimal media were used for other MIC experiments as indicated. Bacteria were grown overnight, and an inoculum of 5 × 10^5^ CFU/mL (final concentration) was prepared. MICs were also determined in the presence and absence of iron salts. M9 medium was used to prepare the inoculum, and four different concentrations of FeSO_4_ and FeCl_3_ (10, 20, 30, and 40 µM) were used.

### Time-dependent killing

Clinical isolate *P. aeruginosa* C0060 was grown overnight in CAMHB, and the OD_600 was_ adjusted to 0.1. The cells were diluted 100-fold in fresh media containing 1 × MIC and 5 × MIC of vacidobactin A or 1 × MIC of tobramycin and were incubated at 37°C. The cells without supplemented antibiotics served as the positive control. Ten microliters of treated cells were spotted onto CAMHB agar plates at four different time points (0 h, 3 h, 6 h, and 12 h), and CFUs were counted. The experiment was conducted in duplicate and repeated three times on separate days. For optical density measurement experiments, serial dilution was conducted with different concentrations of VacA, and OD_600 nm_ was recorded from 0 to 18 h. The experiment was conducted in triplicate, with two biological repeats.

### Generation of *P. aeruginosa* mutants

A *P. aeruginosa* PA14 ∆*pvdA* ∆*pchA* double mutant was generated by allelic exchange. Primers flanking the upstream (~1,000 bp) and downstream (~750 bp) regions of *pvdA* and *pchA* were PCR-amplified from PA14 genomic DNA (Promega Wizard Genomic DNA Purification Kit) with the primers described in Table S4 and gel-extracted with GeneJet Gel Extraction Kit (ThermoFisher).

To make the ∆*pvdA* deletion construct, the upstream fragment was digested with EcoRI and BamHI (FastDigest, ThermoFisher) and then ligated into pEX18Gm digested with the same enzymes (T4 DNA ligase, ThermoFisher). The ligation mixtures were transformed into competent *E. coli* DH5α by heat shock with a recovery period of 3 h in lysogeny broth (LB, BioShop). Cells were plated on LB 1.5% agar containing 15 µg/mL gentamicin supplemented with X-gal for blue-white screening. The plates were incubated at 37°C overnight. White colonies were selected and grown in LB + 15 µg/mL gentamicin. Cells were spun down, and the plasmids were isolated using GeneJet Plasmid Miniprep Kit (ThermoFisher). Plasmids were checked by digestion with EcoRI and BamHI. The plasmid was then digested with BamHI and HindIII and ligated with the digested downstream fragment, and the same checks were repeated.

To generate the ∆*pchA* deletion construct, the upstream and downstream fragments were joined by overlap extension PCR and digested with EcoRI and HindIII. pEX18Gm was digested with the same enzymes, and the fragments were ligated overnight with T4 DNA ligase as outlined above.

Plasmids were transformed into competent *E. coli* SM10 by heat shock with a recovery period of 3 h in LB. The cells were plated on LB 1.5% agar containing 15 µg/mL gentamicin and grown overnight at 37°C. One colony was inoculated in LB + 15 µg/mL gentamicin overnight. PA14 was also inoculated from a single colony into LB. Both cultures were grown overnight at 37°C. SM10 pEX18Gm-*∆pvdA* was mated with PA14 by mixing 500 µL of each overnight culture. Cells were centrifuged, and the supernatant was removed. Cells were resuspended in 50 µL fresh LB, spotted on LB 1.5% agar, and incubated overnight at 37°C. Cells were streaked onto *Pseudomonas* Isolation Agar (PIA, Difco) supplemented with 100 µg/mL gentamicin and incubated overnight at 37°C. Single colonies were streaked onto LB + 15% sucrose (BioShop) and incubated overnight at 37°C. To check for colonies with the correct deletion, 16 colonies were patched onto LB + 15% sucrose and LB + 30 µg/mL gentamicin and incubated overnight at 37°C. Patches that grew on the sucrose plates but not gentamicin plates were colony PCR checked with primers flanking the *pvdA* gene compared to wild-type controls. PA14 ∆*pvdA* was streaked onto LB + 15% sucrose to isolate single colonies, incubated overnight at 37°C, and verified by colony PCR. A single colony was inoculated into LB broth, and the process was repeated to delete *pchA* and generate a siderophore-null *P. aeruginosa* mutant.

### Growth restoration assay between pyoverdine and VacA

The MIC of VacA was determined in M9 medium for a *P. aeruginosa* ∆*pvdA* mutant (unable to synthesize pyoverdine [38]) in the presence and absence of pyoverdine (10 µM; Sigma Aldrich). The difference in the MICs of VacA in the presence and absence of pyoverdine was used to measure competition between the two siderophores.

### Pyoverdine competition assay

Pyoverdine concentration was taken at 10 µM, and its fluorescence was measured with varying concentrations of FeCl_3_. The assay was performed in a black 96-well plate. One microliter each of pyoverdine and FeCl_3_ was added to the wells, and 98 µL of PBS was added; the fluorescence of pyoverdine was measured with excitation/emission of 407/460 nm in a BioTek Neo Synergy plate reader. For the competition assay, pyoverdine and FeCl_3_ were fixed at 10 and 15 µM, respectively, and the concentration of VacA was varied. One microliter of pyoverdine was added to each well first, and then 99 µL containing iron and varying concentrations of VacA was added. The fluorescence was measured as mentioned above (16).

### Measurement of growth and pyoverdine production levels

To determine whether the clinical isolates could produce pyoverdine, we cultured all isolates under iron-limited conditions and assessed pyoverdine production. Initially, the isolates were grown in 150 μL of LB medium in 96-well plates under static conditions at a 37°C incubator for 16–18 h. The next day, 2 μL of the overnight cultures were transferred to 200 μL of iron-limited CAA medium in 96-well plates. The CAA medium was supplemented with 25 mM HEPES buffer, 20 mM NaHCO₃, and 100 μg/mL human apo-transferrin, a potent natural iron chelator. The experiment was performed in triplicate, with ∆*pvdA* as the negative control and PA14 as the positive control. After incubating the plates at 37°C in the dark for 18 h, growth was measured as optical density at 600 nm (OD_600_), and pyoverdine production was quantified as relative fluorescence units (RFUs) using a BioTek Synergy plate reader (excitation at 400 nm and emission at 460 nm). Relative growth and pyoverdine production for each isolate were calculated by normalizing their OD600 and RFU values to the average OD600 and RFU of the reference strains.

### Growth inhibition and pyoverdine fluorescence assays

To evaluate the effect of VacA on bacterial growth and pyoverdine-associated fluorescence, *P. aeruginosa* PA14 wild type, receptor deletion mutants (ΔfpvA, ΔfpvB, and ΔfpvAΔfpvB), and the complemented strain carrying the transporter construct (ΔfpvAΔfpvB + comp A) or empty vector control were cultured under identical conditions. Overnight cultures were diluted to an optimum optical density and inoculated into fresh medium containing increasing concentrations of VacA (0–128 µg/mL). For growth inhibition analysis, bacterial growth was quantified by measuring optical density at 600 nm (OD₆₀₀) after incubation. For fluorescence analysis, cultures grown under the same experimental conditions were subjected to pyoverdine fluorescence measurement using a microplate reader. RFUs were measured with excitation/emission of 407/460 nm in BioTek Neo Synergy plate reader. Fluorescence values were used to assess whether VacA altered pyoverdine-associated fluorescence across different receptor backgrounds.

### Preparation of overexpression strains

Primers were used to amplify full-length *fiuA* (*PA14_06160*) plus 22 bp upstream of the start codon encompassing its putative translational start site. The sequence encoding the N-terminus was also amplified (417 bp after the start codon) with a 20 bp extension for the overlapping region of the *V. paradoxus* TonB-dependent transporter in the BGC predicted by antiSMASH. Similarly, the overlapping region from the *V. paradoxus* TonB-dependent transporter was amplified from *V. paradoxus* genomic DNA to the stop codon. The N-terminus of FiuA was fused to the barrel and plug domain of the *V. paradoxus* TonB-dependent transporter by overlap extension PCR. The chimeric transporter was expressed from the arabinose-inducible plasmid, pHERD20T (39). The full-length fusion fragment and *fiuA* were digested with EcoRI and HindIII (FastDigest, ThermoFisher), and ligated into pHERD20T, also digested with EcoRI and HindIII. Ligations and selection protocols were identical except for the blue-white screening method; plates were also supplemented with 100 µL of 20% arabinose in LB. Correct plasmids were electroporated into PA14 ∆*pvdA* ∆*pchA* with a recovery period of 1 h and plated onto LB 1.5% agar + 200 µg/mL carbenicillin. Plates were incubated overnight at 37°C, and single colonies were selected for MIC assays.

### Checkerboard synergy assays

The synergy assay for VacA and thiostrepton was conducted using a checkerboard format in 96-well plates. An 8 × 9 checkerboard layout was used, with the concentration of one compound increasing along the y-axis and the concentration of the second compound increasing along the x-axis. The assay was performed in CAMHB. Each well had a final volume of 100 µL, and serial dilutions of the compounds were prepared to achieve a range of concentrations for each drug. Each well was inoculated with *P. aeruginosa* culture at a final inoculum of 5 × 10^5^ CFU/mL. The plates were covered to prevent evaporation and incubated at 37°C for 16–18 h. Following incubation, the optical density at 600 nm (OD_600_) was measured using a BioTek microplate reader. At least three biological replicates were performed for each combination. Data were analyzed in Excel and plotted against compound concentrations using Prism software (GraphPad).

### Combination synergy assay

To further evaluate the synergistic effect of VacA and thiostrepton, a combination assay was performed using 21 clinical *P. aeruginosa* isolates. Freshly streaked plates were used to inoculate 200 µL of CaMHB broth, followed by incubation for 16 h at 37°C. After overnight incubation, 6 µL of the culture was transferred into 144 µL of fresh CaMHB and incubated for 2–4 h at 37°C to reach mid-log phase. For the combination assay, each well contained 2 µL of the 2–4 h subculture of *P. aeruginosa*. For the assay, 2 µL of a combination of VacA (8 µg/mL) and thiostrepton (0.5 µg/mL) and 146 µL of cation-adjusted MHB media, for a total volume of 150 µL per well. The plates were incubated overnight at 37°C. After 16 h, the OD_600_ was measured to assess bacterial growth inhibition using a plate reader. Each experiment was conducted in triplicate and with three biological repeats to ensure reproducibility.

## Data Availability

This Whole Genome Shotgun project of Variovorax paradoxus MST 127 has been deposited at DDBJ/ENA/GenBank under the accession JBSXPN000000000. The version described in this paper is version JBSXPN010000000.

## SUPPLEMENTAL MATERIAL

Supplemental tables and figures spectrum.01240-26-s0001.pdf Tables S1 to S19 and Fig. S1 to S6.mbio

ASM does not own the copyrights to Supplemental Material that may be linked to, or accessed through, an article. The authors have granted ASM a non-exclusive, world-wide license to publish the Supplemental Material files. Please contact the corresponding author directly for reuse.

## References

[1] Moradali MF, Ghods S, Rehm BHA. 2017. *Pseudomonas aeruginosa* lifestyle: a paradigm for adaptation, survival, and persistence. Front Cell Infect Microbiol 7:39. doi:10.3389/fcimb.2017.00039 PMC5310132 28261568

[2] Stover CK, Pham XQ, Erwin AL, Mizoguchi SD, Warrener P, Hickey MJ, Brinkman FS, Hufnagle WO, Kowalik DJ, Lagrou M, et al.. 2000. Complete genome sequence of *Pseudomonas aeruginosa* PAO1, an opportunistic pathogen. Nature 406:959–964. doi:10.1038/35023079 10984043

[3] Qin SG, Xiao W, Zhou CM, Pu QQ, Deng X, Lan LF, Liang HH, Song XR, Wu M. 2022. *Pseudomonas aeruginosa*: pathogenesis, virulence factors, antibiotic resistance, interaction with host, technology advances and emerging therapeutics. Sig Transduct Target Ther 7. doi:10.1038/s41392-022-01056-1 PMC9233671 35752612

[4] Tuon FF, Dantas LR, Suss PH, Tasca Ribeiro VS. 2022. Pathogenesis of the *Pseudomonas aeruginosa* biofilm: a review. Pathogens 11:300. doi:10.3390/pathogens11030300 PMC8950561 35335624

[5] WHO. 2024. Antibacterial agents in clinical and preclinical development: an overview and analysis. World Health Organization, Geneva.

[6] Wei X, Zhou D, Xu C, Chen P, Chen S, Cheng Z, Jin Y, Jin S, Wu W. 2024. Murepavadin enhances the killing efficacy of ciprofloxacin against *Pseudomonas aeruginosa* by Inhibiting drug efflux. Antibiotics (Basel) 13:810. doi:10.3390/antibiotics13090810 PMC11429200 39334985

[7] Cook MA, Pallant D, Ejim L, Sutherland AD, Wang X, Johnson JW, McCusker S, Chen X, George M, Chou S, Koteva K, Wang W, Hobson C, Hackenberger D, Waglechner N, Ejim O, Campbell T, Medina R, MacNeil LT, Wright GD. 2023. Lessons from assembling a microbial natural product and pre-fractionated extract library in an academic laboratory. J Ind Microbiol Biotechnol 50:kuad042. doi:10.1093/jimb/kuad042 PMC10724011 38052426

[8] Butaitė E, Baumgartner M, Wyder S, Kümmerli R. 2017. Siderophore cheating and cheating resistance shape competition for iron in soil and freshwater *Pseudomonas* communities. Nat Commun 8:414. doi:10.1038/s41467-017-00509-4 PMC5583256 28871205

[9] Jangra M, Travin DY, Aleksandrova EV, Kaur M, Darwish L, Koteva K, Klepacki D, Wang W, Tiffany M, Sokaribo A, Chen X, Deng Z, Tao M, Coombes BK, Vázquez-Laslop N, Polikanov YS, Mankin AS, Wright GD. 2025. A broad-spectrum lasso peptide antibiotic targeting the bacterial ribosome. Nature 640:1022–1030. doi:10.1038/s41586-025-08723-7 PMC12497486 40140562

[10] Kurth C, Schieferdecker S, Athanasopoulou K, Seccareccia I, Nett M. 2016. Variochelins, lipopeptide siderophores from *Variovorax boronicumulans* discovered by genome mining. J Nat Prod 79:865–872. doi:10.1021/acs.jnatprod.5b00932 27023373

[11] Robertson AW, McCarville NG, MacIntyre LW, Correa H, Haltli B, Marchbank DH, Kerr RG. 2018. Isolation of imaqobactin, an amphiphilic siderophore from the arctic marine bacterium variovorax species RKJM285. J Nat Prod 81:858–865. doi:10.1021/acs.jnatprod.7b00943 29616814

[12] Johnston CW, Skinnider MA, Wyatt MA, Li X, Ranieri MRM, Yang L, Zechel DL, Ma B, Magarvey NA. 2015. An automated genomes-to-natural products platform (GNP) for the discovery of modular natural products. Nat Commun 6:8421. doi:10.1038/ncomms9421 PMC4598715 26412281

[13] Hardy CD, Butler A. 2018. β-Hydroxyaspartic acid in siderophores: biosynthesis and reactivity. J Biol Inorg Chem 23:957–967. doi:10.1007/s00775-018-1584-2 29946978

[14] Blin K, Shaw S, Steinke K, Villebro R, Ziemert N, Lee SY, Medema MH, Weber T. 2019. antiSMASH 5.0: updates to the secondary metabolite genome mining pipeline. Nucleic Acids Res 47:W81–W87. doi:10.1093/nar/gkz310 PMC6602434 31032519

[15] Liberati NT, Urbach JM, Miyata S, Lee DG, Drenkard E, Wu G, Villanueva J, Wei T, Ausubel FM. 2006. An ordered, nonredundant library of *Pseudomonas aeruginosa* strain PA14 transposon insertion mutants. Proc Natl Acad Sci USA 103:2833–2838. doi:10.1073/pnas.0511100103 PMC1413827 16477005

[16] Xiao R, Kisaalita WS. 1998. Fluorescent pseudomonad pyoverdines bind and oxidize ferrous ion. Appl Environ Microbiol 64:1472–1476. doi:10.1128/AEM.64.4.1472-1476.1998 PMC106172 9575133

[17] Ranieri MRM, Chan DCK, Yaeger LN, Rudolph M, Karabelas-Pittman S, Abdo H, Chee J, Harvey H, Nguyen U, Burrows LL. 2019. Thiostrepton hijacks pyoverdine receptors to inhibit growth of *Pseudomonas aeruginosa*. Antimicrob Agents Chemother 63:e00472-19. doi:10.1128/AAC.00472-19 PMC6709454 31262758

[18] Lamont IL, Beare PA, Ochsner U, Vasil AI, Vasil ML. 2002. Siderophore-mediated signaling regulates virulence factor production in *Pseudomonasaeruginosa*. The National Academy of Sciences 99:7072–7077. doi:10.1073/pnas.092016999 PMC124530 11997446

[19] Shen J, Meldrum A, Poole K. 2002. FpvA receptor involvement in pyoverdine biosynthesis in *Pseudomonas aeruginosa*. J Bacteriol 184:3268–3275. doi:10.1128/JB.184.12.3268-3275.2002 PMC135083 12029043

[20] Kang D, Revtovich AV, Chen Q, Shah KN, Cannon CL, Kirienko NV. 2019. Pyoverdine-dependent virulence of *Pseudomonas aeruginosa* isolates from cystic fibrosis patients. Front Microbiol 10:2048. doi:10.3389/fmicb.2019.02048 PMC6743535 31551982

[21] Smith EE, Buckley DG, Wu Z, Saenphimmachak C, Hoffman LR, D’Argenio DA, Miller SI, Ramsey BW, Speert DP, Moskowitz SM, Burns JL, Kaul R, Olson MV. 2006. Genetic adaptation by *Pseudomonas aeruginosa* to the airways of cystic fibrosis patients. The National Academy of Sciences 103:8487–8492. doi:10.1073/pnas.0602138103 PMC1482519 16687478

[22] Pál C, Papp B, Lázár V. 2015. Collateral sensitivity of antibiotic-resistant microbes. Trends Microbiol 23:401–407. doi:10.1016/j.tim.2015.02.009 PMC5958998 25818802

[23] Kümmerli R. 2023. Iron acquisition strategies in pseudomonads: mechanisms, ecology, and evolution. Biometals 36:777–797. doi:10.1007/s10534-022-00480-8 PMC10393863 36508064

[24] Minandri F, Imperi F, Frangipani E, Bonchi C, Visaggio D, Facchini M, Pasquali P, Bragonzi A, Visca P. 2016. Role of iron uptake systems in *Pseudomonas aeruginosa* virulence and airway infection. Infect Immun 84:2324–2335. doi:10.1128/IAI.00098-16 PMC4962624 27271740

[25] Kang D, Kirienko NV. 2018. Interdependence between iron acquisition and biofilm formation in *Pseudomonas aeruginosa*. J Microbiol 56:449–457. doi:10.1007/s12275-018-8114-3 PMC6221862 29948830

[26] Ganne G, Brillet K, Basta B, Roche B, Hoegy F, Gasser V, Schalk IJ. 2017. Iron release from the siderophore pyoverdine in *Pseudomonas aeruginosa* involves three new actors: FpvC, FpvG, and FpvH. ACS Chem Biol 12:1056–1065. doi:10.1021/acschembio.6b01077 28192658

[27] Albrecht-Gary A-M, Blanc S, Rochel N, Ocaktan AZ, Abdallah MA. 1994. Bacterial iron transport: coordination properties of pyoverdin PaA, a peptidic siderophore of *Pseudomonas aeruginosa*. Inorg Chem 33:6391–6402. doi:10.1021/ic00104a059

[28] Haedar JR, Yoshimura A, Wakimoto T. 2024. New variochelins from soil-isolated Variovorax sp. Beilstein J Org Chem 20:692–700. doi:10.3762/bjoc.20.63 PMC10999976 38590537

[29] Cornelis P. 2010. Iron uptake and metabolism in pseudomonads. Appl Microbiol Biotechnol 86:1637–1645. doi:10.1007/s00253-010-2550-2 20352420

[30] Chan DCK, Josts I, Koteva K, Wright GD, Tidow H, Burrows LL. 2023. Interactions of TonB-dependent transporter FoxA with siderophores and antibiotics that affect binding, uptake, and signal transduction. Proc Natl Acad Sci USA 120:e2221253120. doi:10.1073/pnas.2221253120 PMC10120069 37043535

[31] Chan DCK, Burrows LL. 2021. Thiocillin and micrococcin exploit the ferrioxamine receptor of *Pseudomonas aeruginosa* for uptake. J Antimicrob Chemother 76:2029–2039. doi:10.1093/jac/dkab124 33907816

[32] Poole K, Young L, Neshat S. 1990. Enterobactin-mediated iron transport in *Pseudomonas aeruginosa*. J Bacteriol 172:6991–6996. doi:10.1128/jb.172.12.6991-6996.1990 PMC210819 2174865

[33] Chan DCK, Guo I, Burrows LL. 2020. Forging new antibiotic combinations under iron-limiting conditions. Antimicrob Agents Chemother 64:e01909-19. doi:10.1128/AAC.01909-19 PMC7038247 31907180

[34] Neumann W, Sassone-Corsi M, Raffatellu M, Nolan EM. 2018. Esterase-catalyzed siderophore hydrolysis activates an enterobactin-ciprofloxacin conjugate and confers targeted antibacterial activity. J Am Chem Soc 140:5193–5201. doi:10.1021/jacs.8b01042 PMC5921047 29578687

[35] Louden BC, Haarmann D, Lynne AM. 2011. Use of blue agar CAS assay for siderophore detection. J Microbiol Biol Educ 12:51–53. doi:10.1128/jmbe.v12i1.249 PMC3577196 23653742

[36] Fujii K, Ikai Y, Mayumi T, Oka H, Suzuki M, Harada K. 1997. A nonempirical method using LC/MS for determination of the absolute configuration of constituent amino acids in a peptide: elucidation of limitations of marfey’s method and of its separation mechanism. Anal Chem 69:3346–3352. doi:10.1021/ac9701795

[37] Ejim L, Farha MA, Falconer SB, Wildenhain J, Coombes BK, Tyers M, Brown ED, Wright GD. 2011. Combinations of antibiotics and nonantibiotic drugs enhance antimicrobial efficacy. Nat Chem Biol 7:348–350. doi:10.1038/nchembio.559 21516114

[38] Chan DCK, Burrows LL. 2023. *Pseudomonas aeruginosa* FpvB is a high-affinity transporter for xenosiderophores ferrichrome and ferrioxamine B. mBio 14:e0314922. doi:10.1128/mbio.03149-22 PMC9973354 36507834

[39] Qiu D, Damron FH, Mima T, Schweizer HP, Yu HD. 2008. PBAD-based shuttle vectors for functional analysis of toxic and highly regulated genes in *Pseudomonas* and *Burkholderia* spp. and other bacteria. Appl Environ Microbiol 74:7422–7426. doi:10.1128/AEM.01369-08 PMC2592904 18849445

