# Supplementary material for: Iron starvation induced by vacidobactin A impairs the growth of pyoverdine-deficient Pseudomonas aeruginosa
Source: Microbiol Spectr. 2026 Aug 26;14(10):e01240-26. doi: 10.1128/spectrum.01240-26 (PMC13689467; doi:10.1128/spectrum.01240-26)
Supplement: Supplementary file 1 [file spectrum.01240-26-s0001.pdf]

SUPPLEMENTARY INFORMATION

Iron starvation induced by vacidobactin A impairs the growth of pyoverdine-deficient *P. aeruginosa*

Manpreet Kaur[1,2,3], Derek C. K. Chan[1,2,3], Hodan Wardere[1,2,3], Wenliang Wang[1,2,3], Manoj Jangra[1,2,3], Allison. K. Guitor[1,2,3], Kalinka K. Koteva[1,2,3], Lori L. Burrows[1,2,3], Gerard D Wright[1,2,3,#]

[1] David Braley Centre for Antibiotics Discovery, McMaster University, Hamilton, Ontario, Canada.
[2] M.G. DeGroote Institute for Infectious Disease Research, McMaster University, Hamilton, Ontario, Canada.
[3] Department of Biochemistry and Biomedical Sciences, McMaster University, Hamilton, Ontario, Canada.

[#]correspondence to: wrightge@mcmaster.ca

SUPPLEMENTARY FIGURES

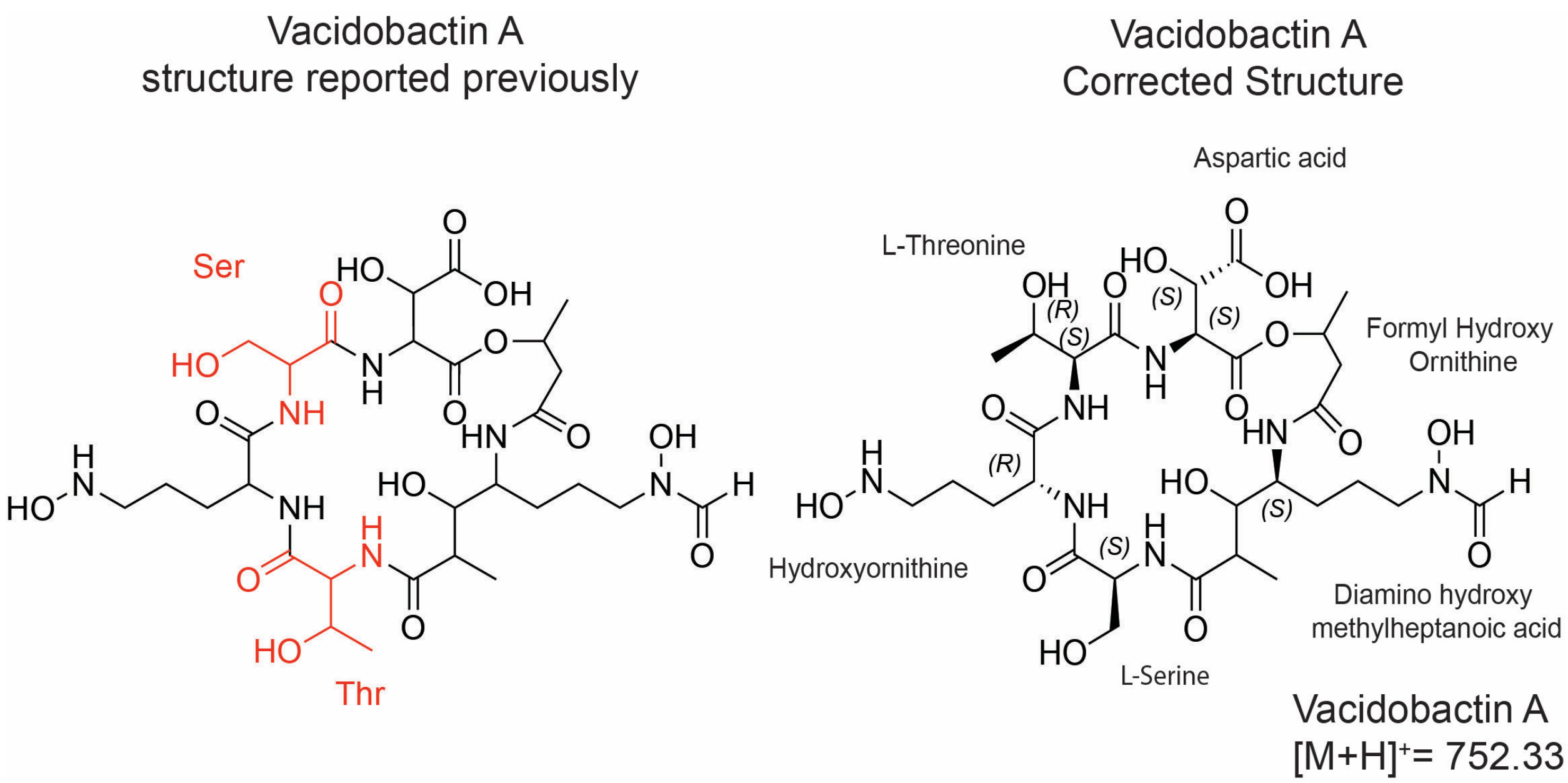

Figure S1. Structural differences between Vacidobactin A (previously reported) and the corrected Vacidobactin A.

Key structural variations are highlighted in red in the previously reported Vacidobactin A. In the corrected Vacidobactin A, a peptide bond is formed between β-OHAsp and threonine at position 2. In contrast, the previously reported Vacidobactin A features a serine at position 2, shown in red

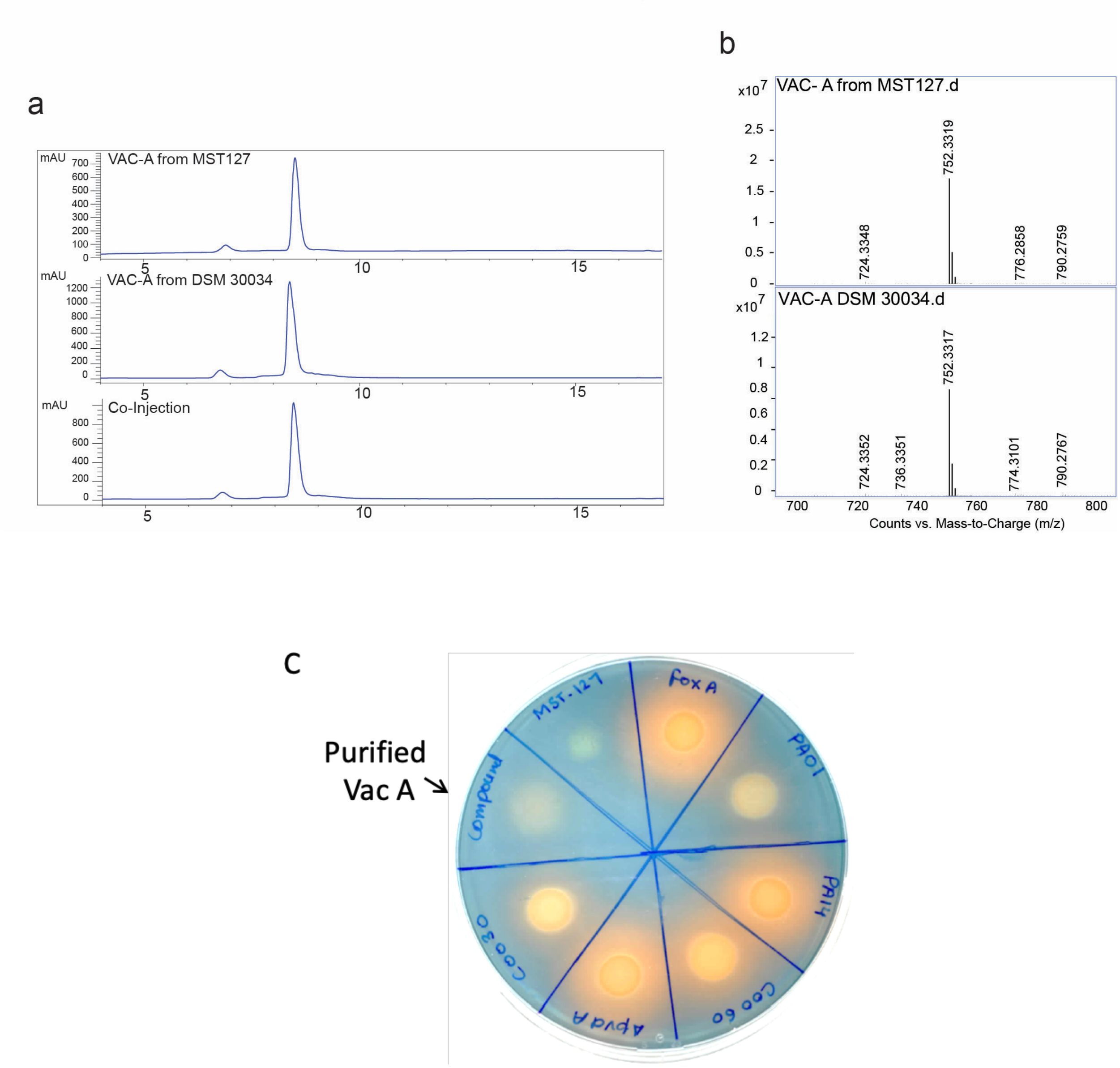

Figure S2. (a) HPLC chromatograms of purified Vac A from *Variovorax* strains MST 127 and DSM 30034, along with their co-injection, demonstrate that they are identical molecules. (b) Mass spectra showing the $[M+H]^+$ ion at m/z 752.33 for VAC A from both strains. (C) Chrome azurol S (CAS) assay using purified VacA, compared with cells of different strains that produce potential iron chelators.

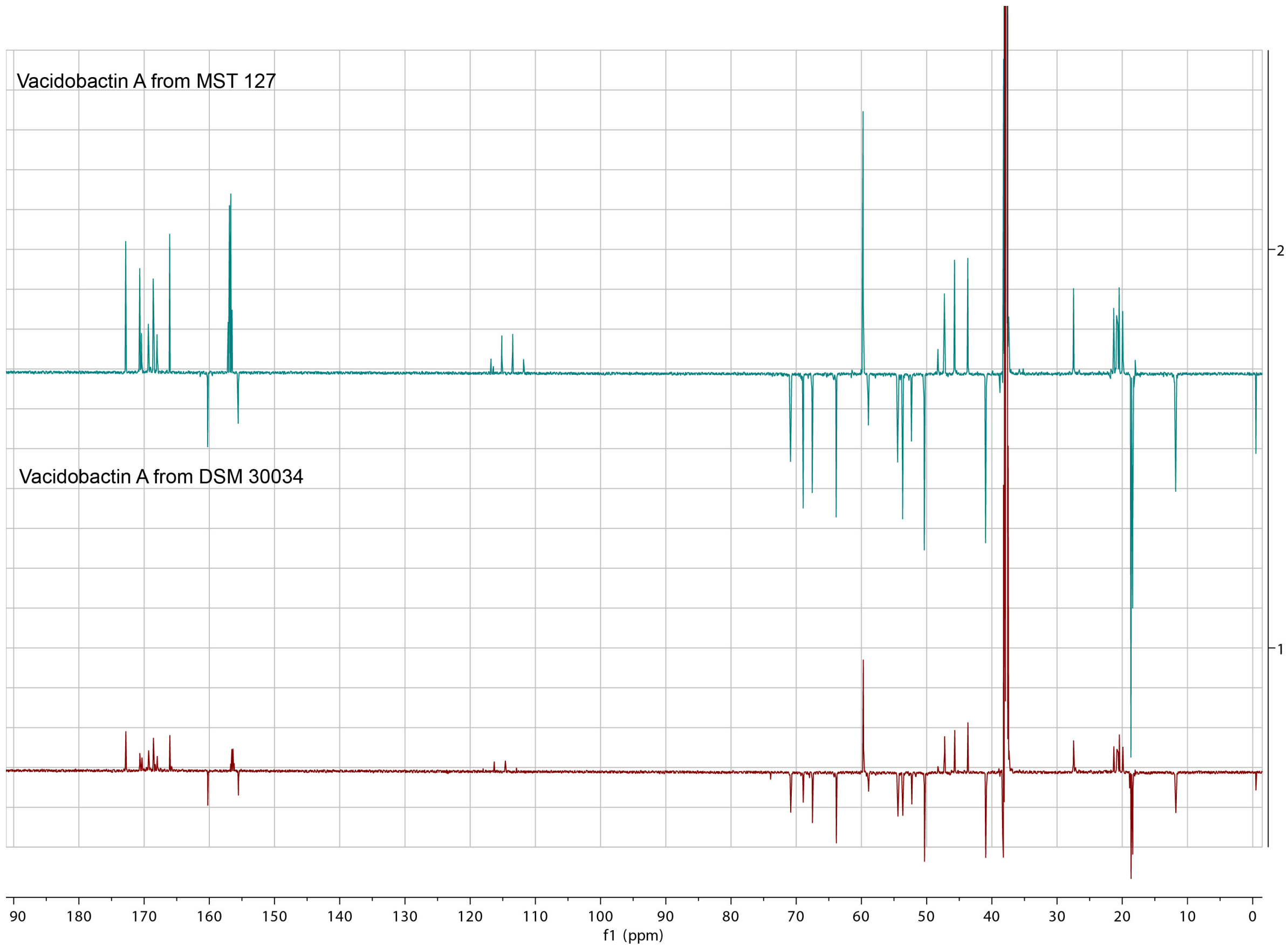

Figure S3. ^{1}H NMR spectrum of VacA comparison from MST 127 and DSM 30034

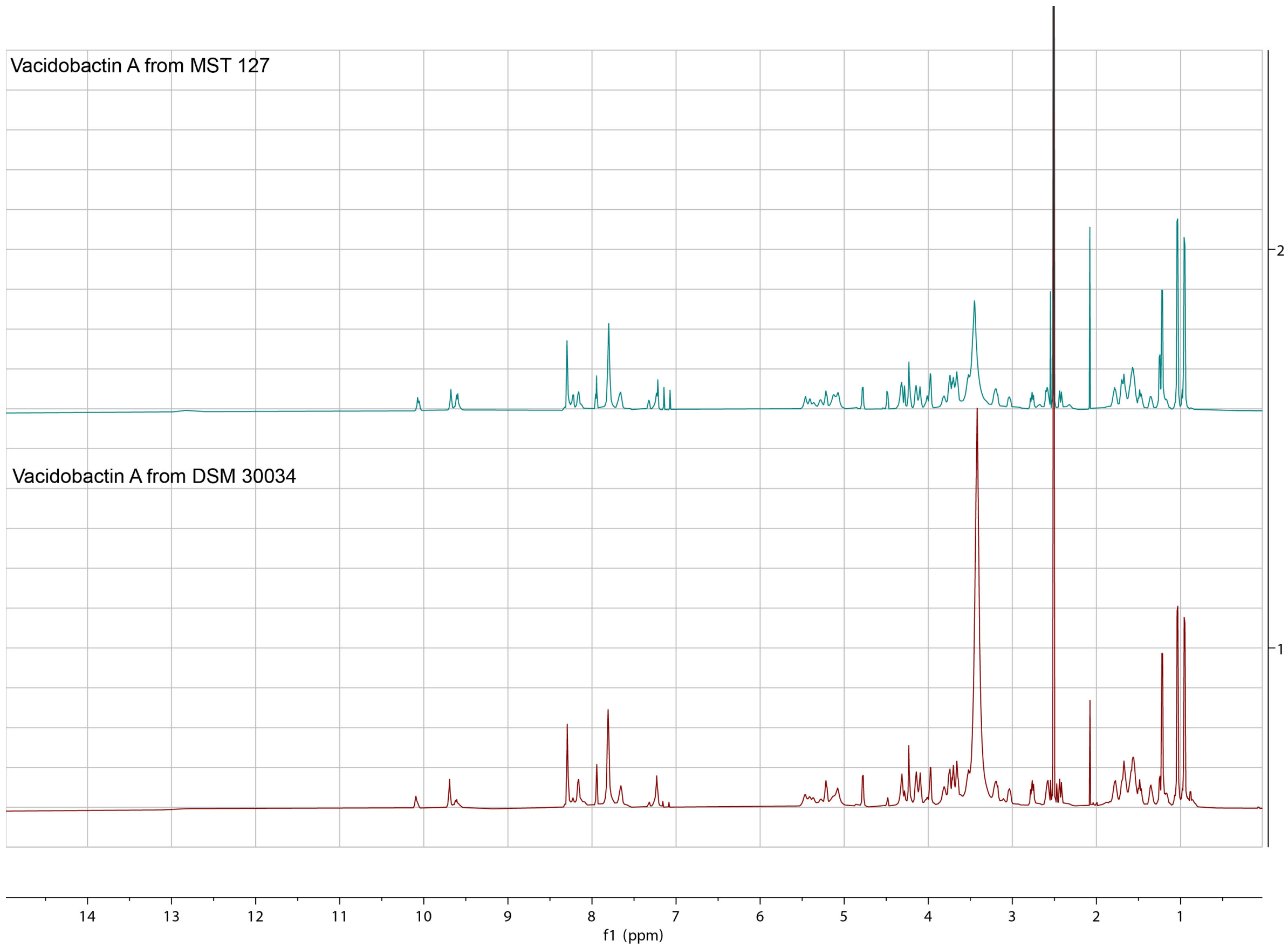

Figure S4. ^{1}H NMR of Dept-Q spectrum of VacA comparison 1 from MST 127 and DSM 30034

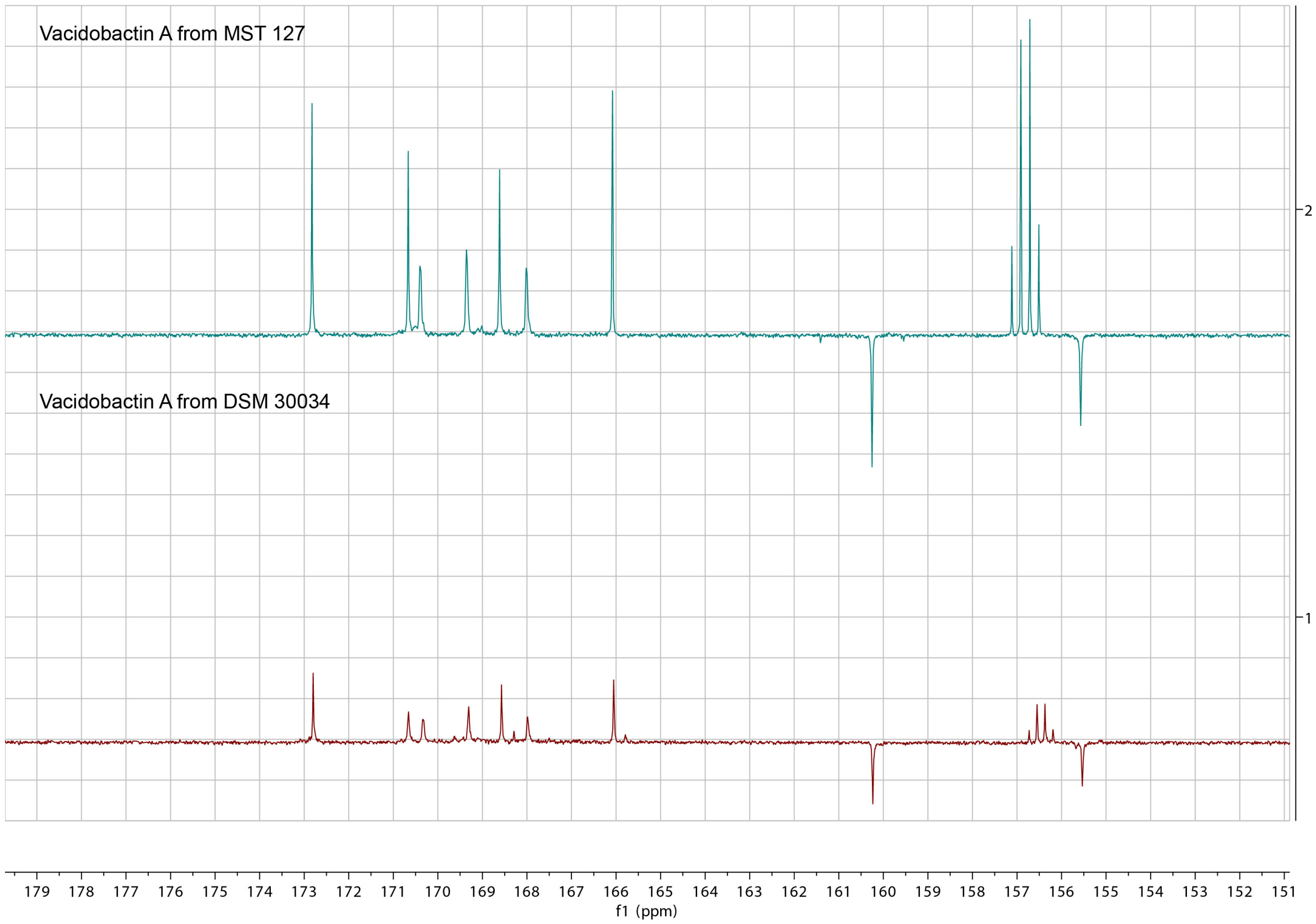

Figure S5. ^{1}H NMR of Dept-Q spectrum of VacA comparison 2 from MST 127 and DSM 30034

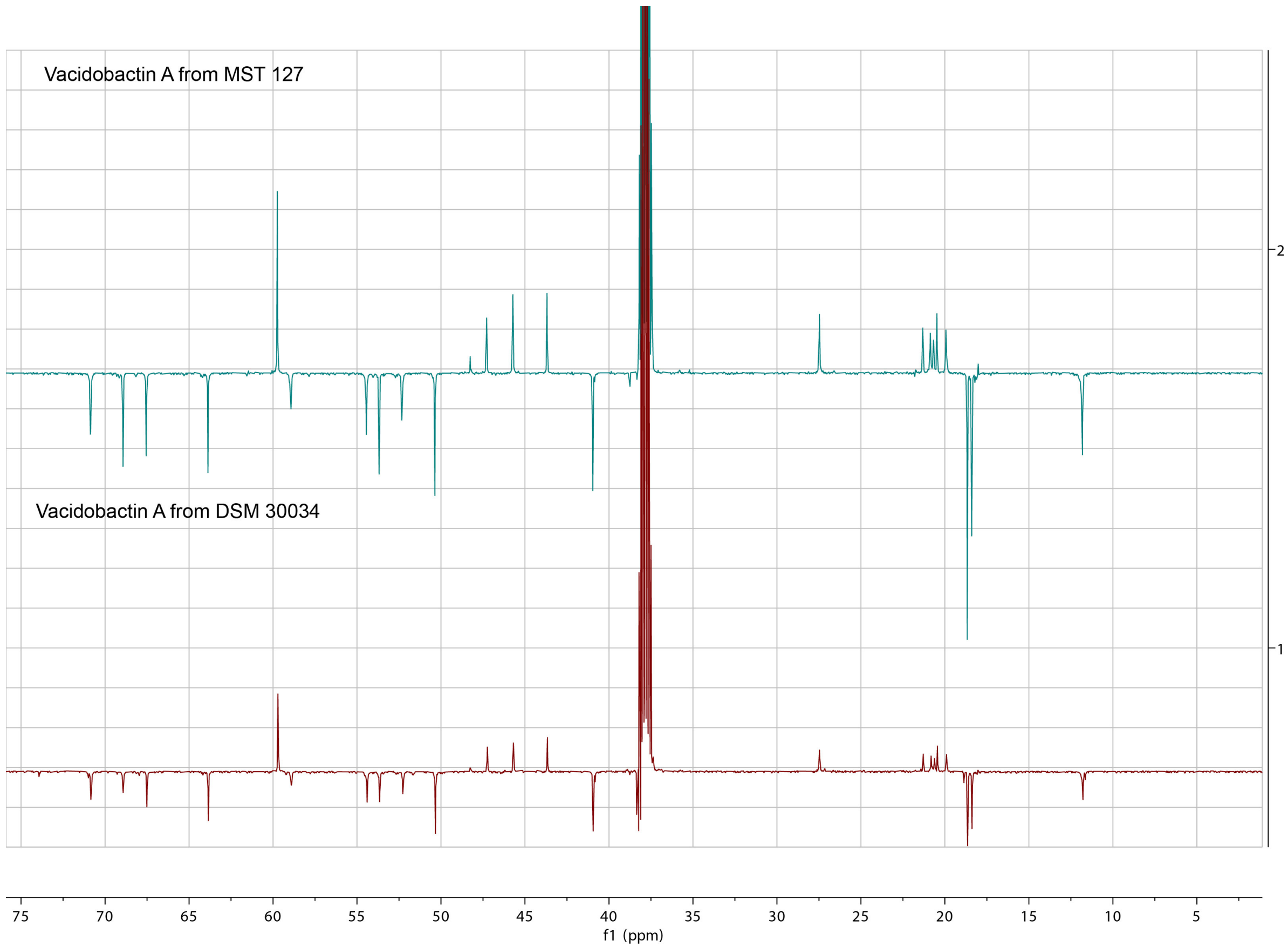

Figure S6. ^{1}H NMR of Dept-Q spectrum of VacA comparison 3 from MST 127 and DSM 30034

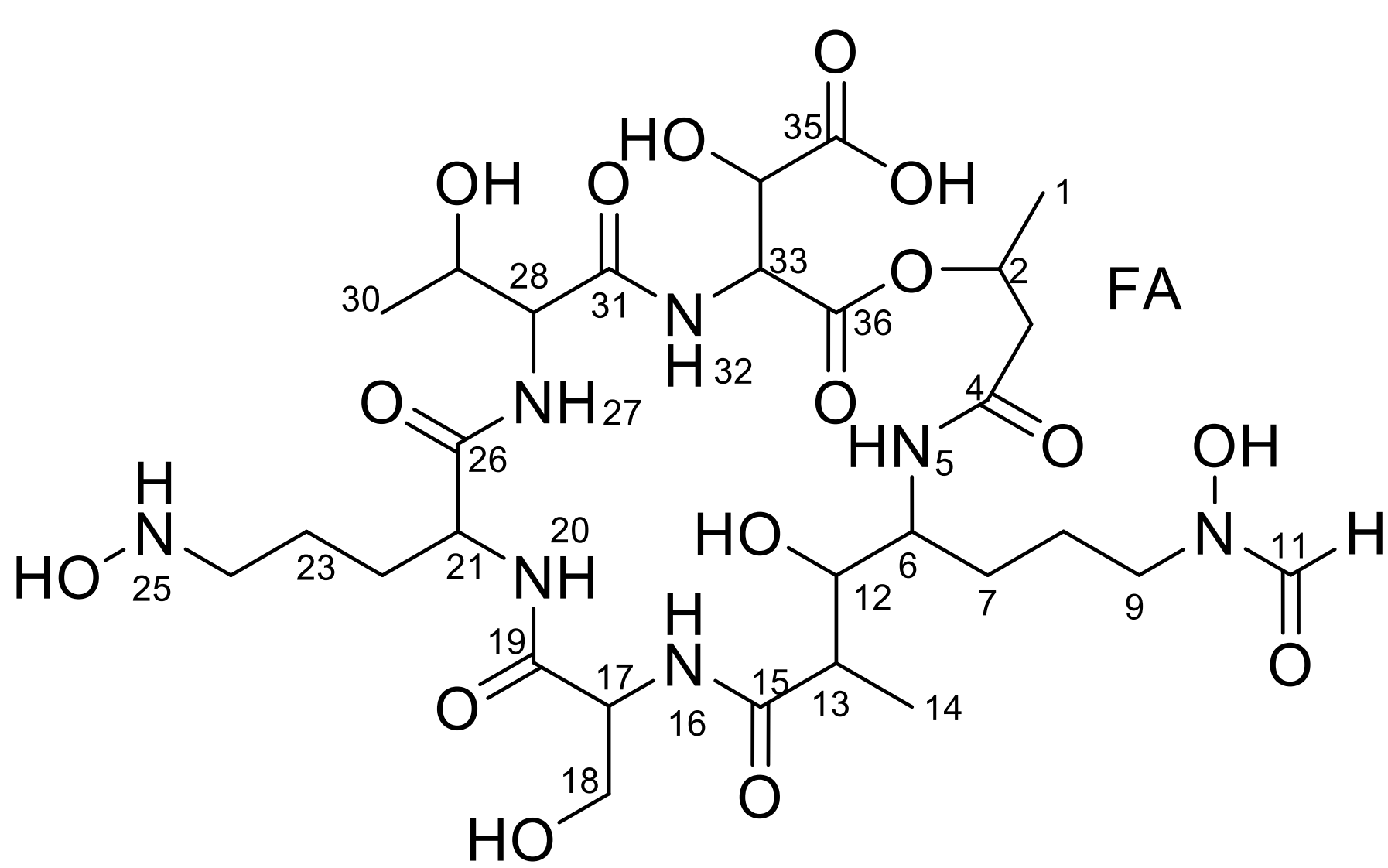

Figure S7. Structural assignments of Vacidobactin A from MST127

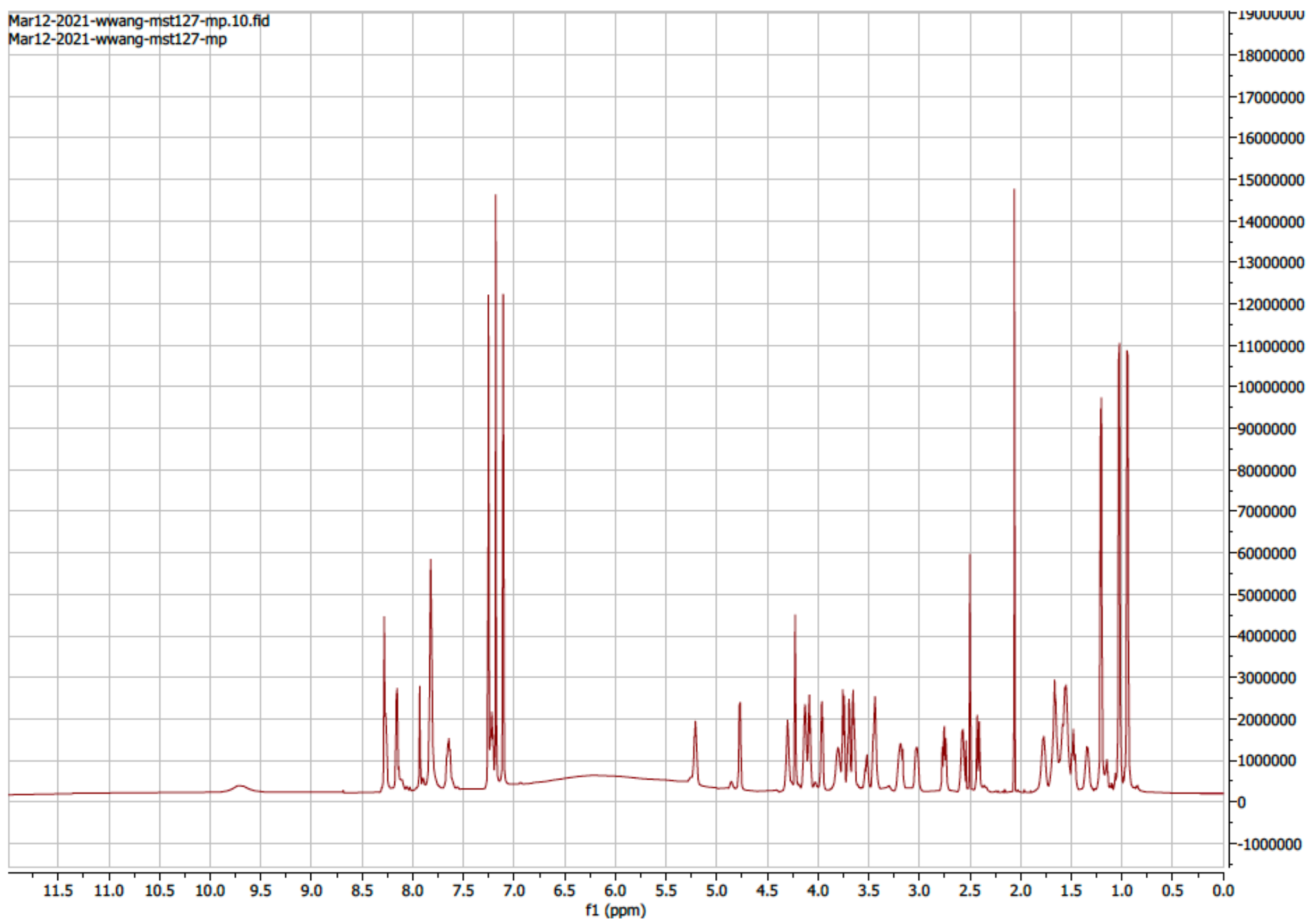

Figure S8. ^{1}H NMR spectrum of VacA from MST127

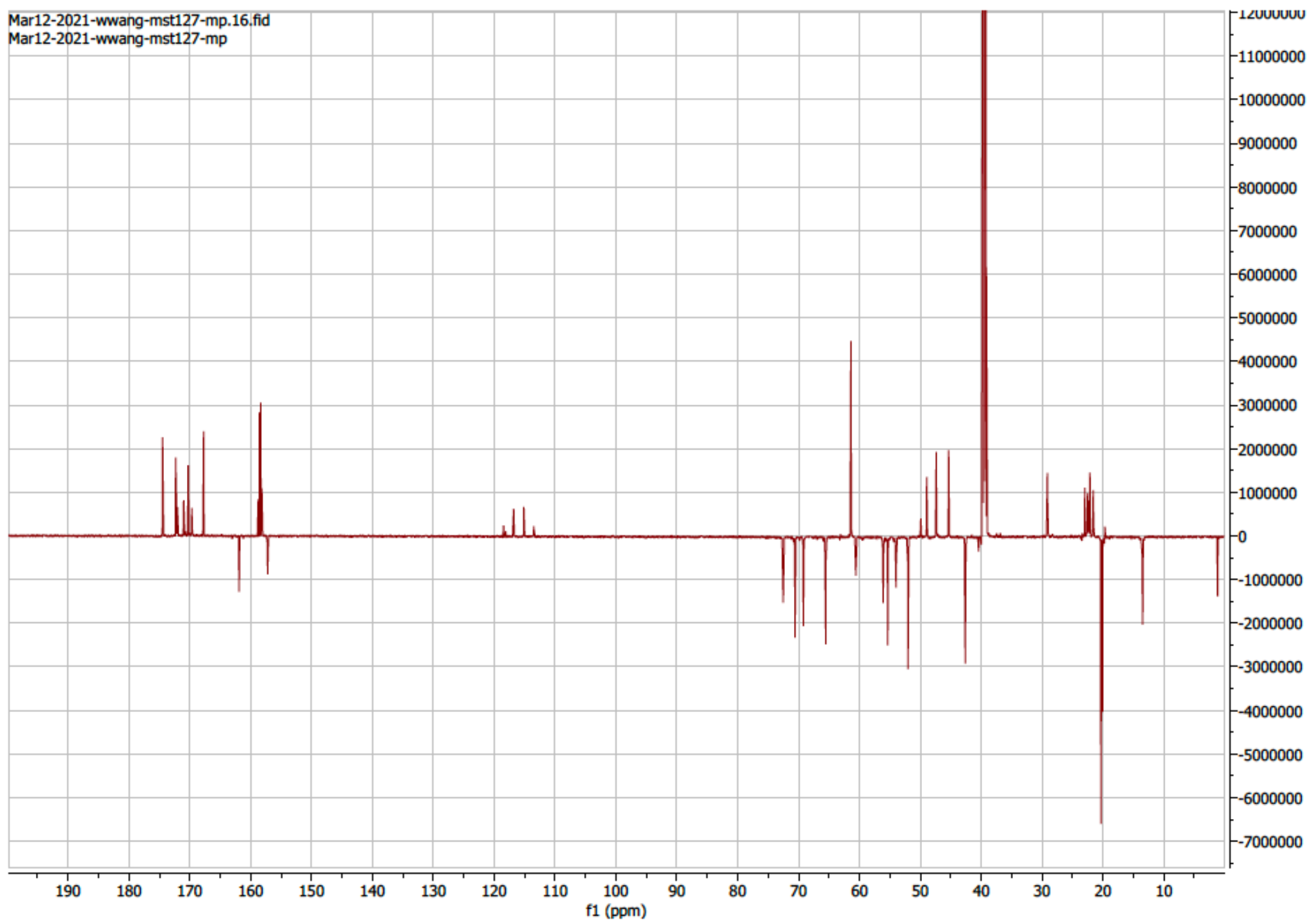

Figure S9. ^{13}C DEPTQ NMR spectrum of VacA from MST127

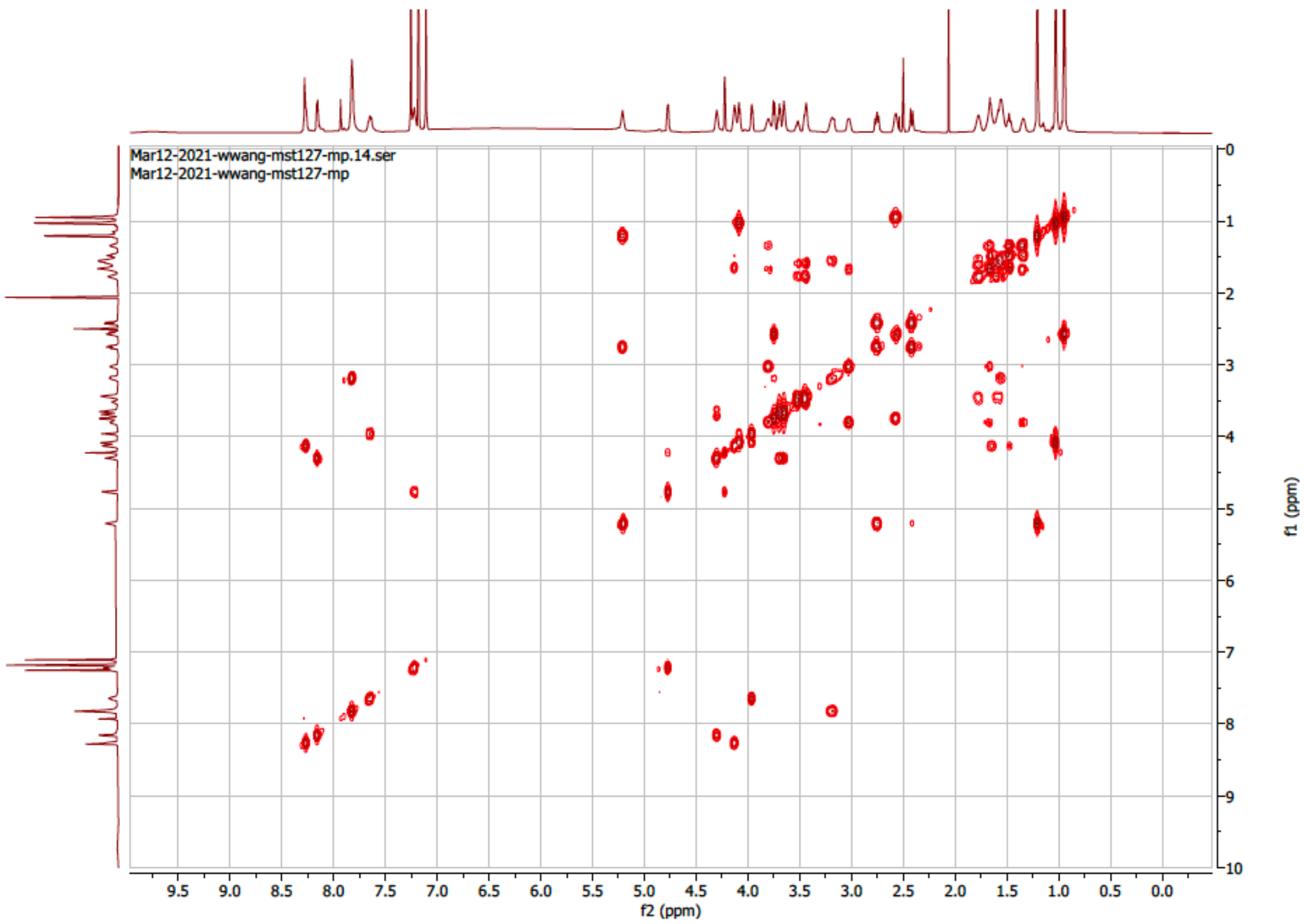

Figure S10. ^{1}H-^{1}H COESY NMR spectrum of VacA from MST127

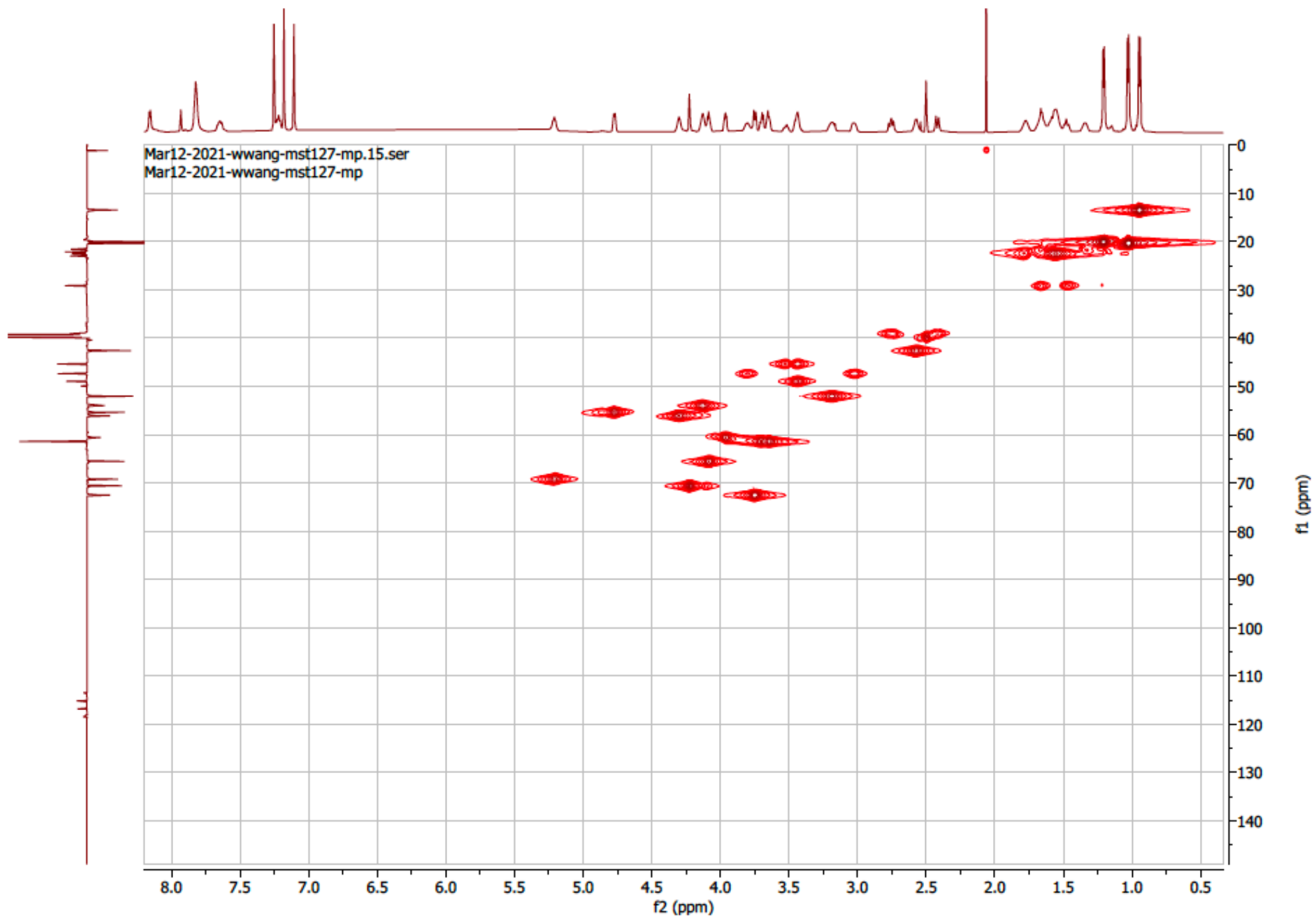

Figure S11. ^{1}H-^{13}C HSQC NMR spectrum of VacA from MST127

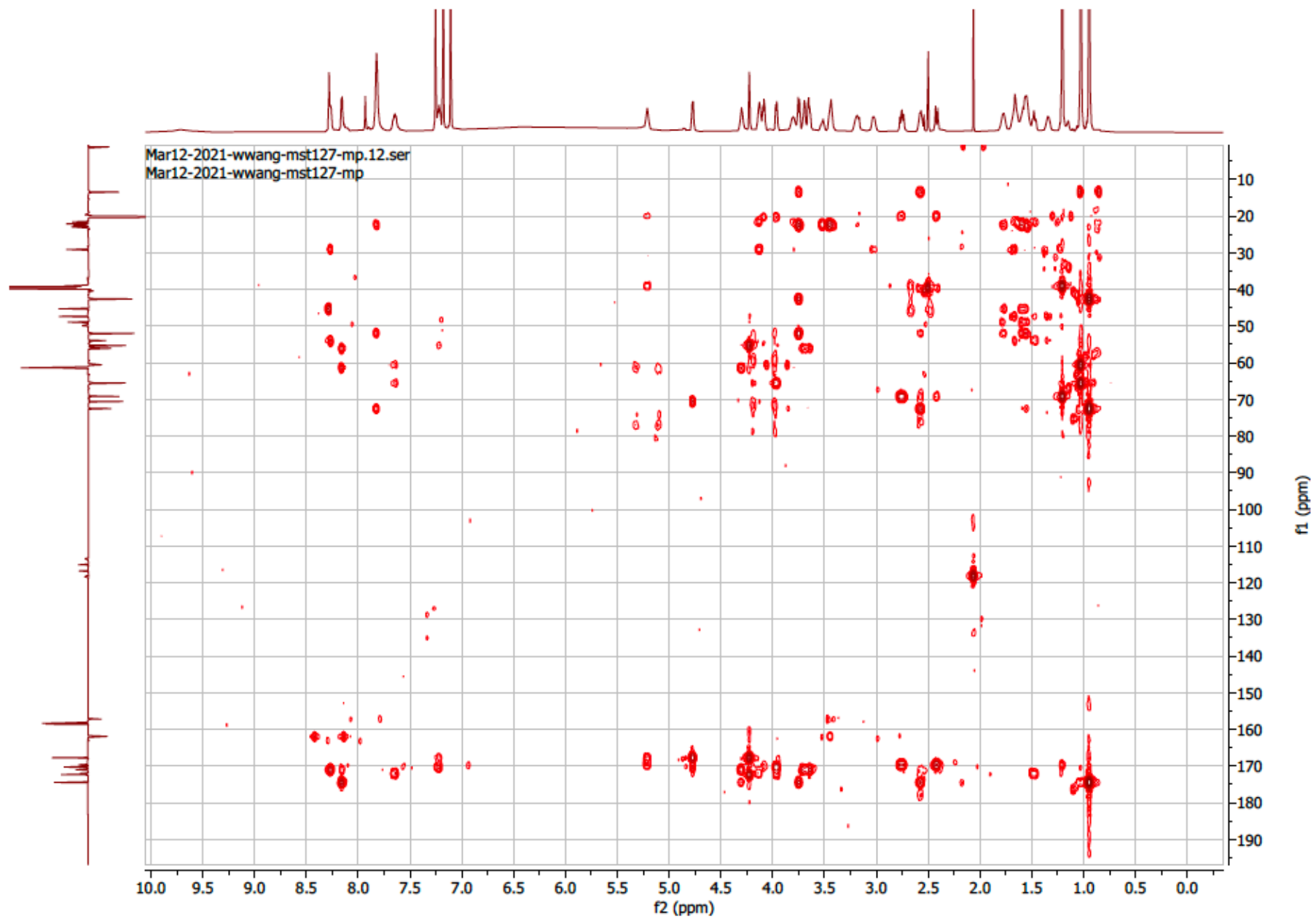

Figure S12.^{1}H-^{13}C HMBC NMR spectrum of VacA from MST127

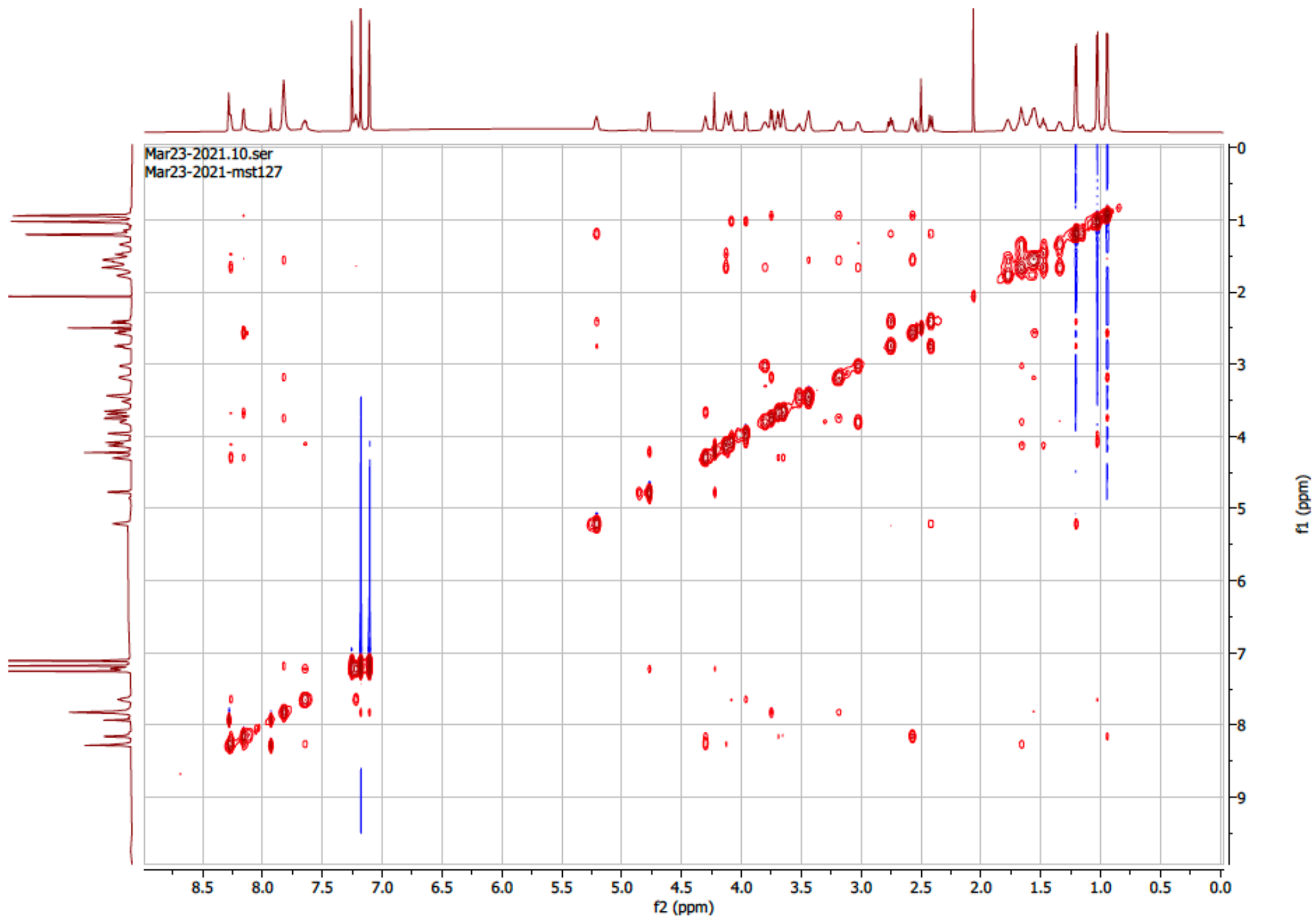

Figure S13. NOESY-NMR spectrum of VacA from MST127

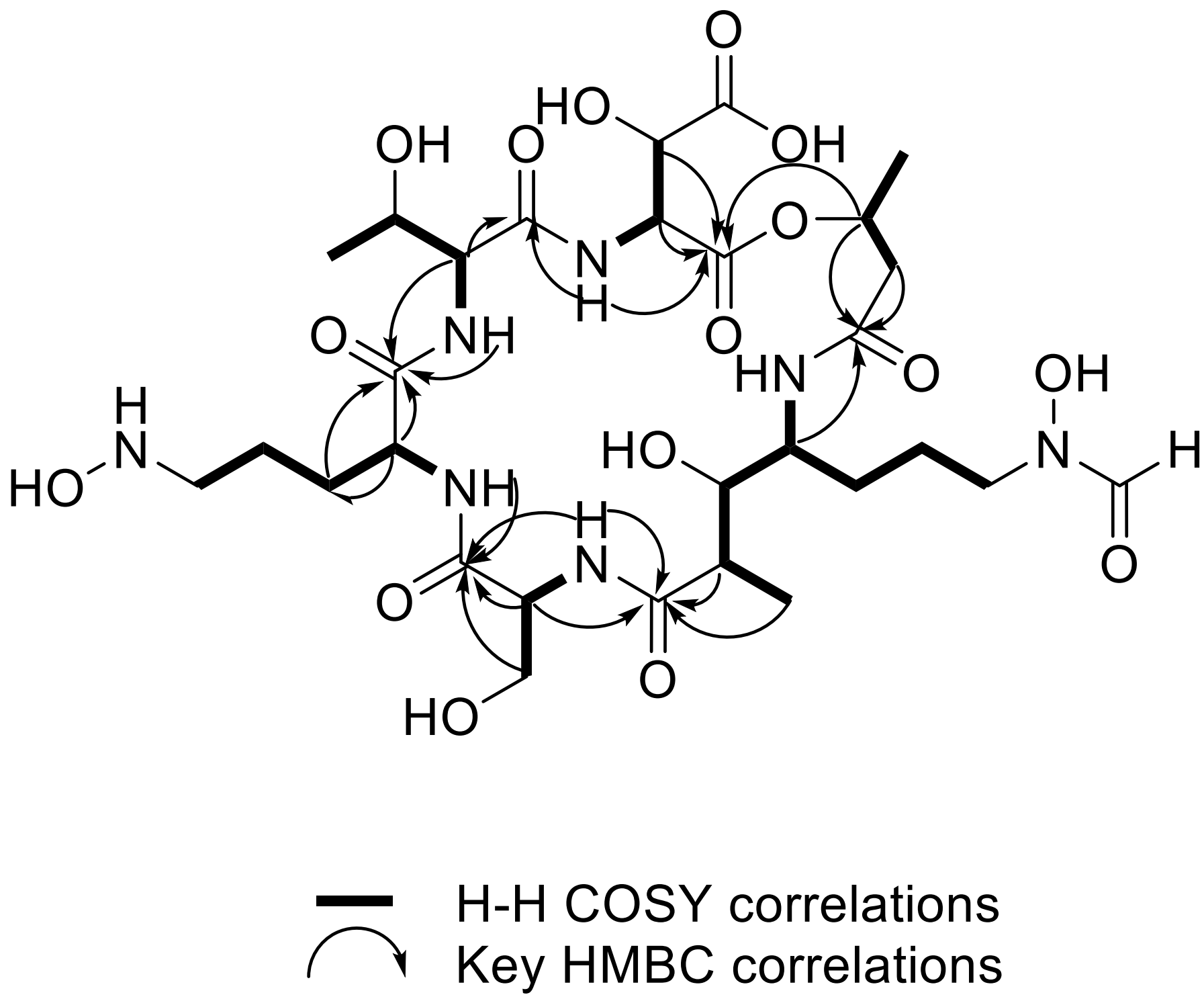

Figure S14. Key COSY- and HMBC-NMR correlations used for the structural elucidation of VacA from MST127.

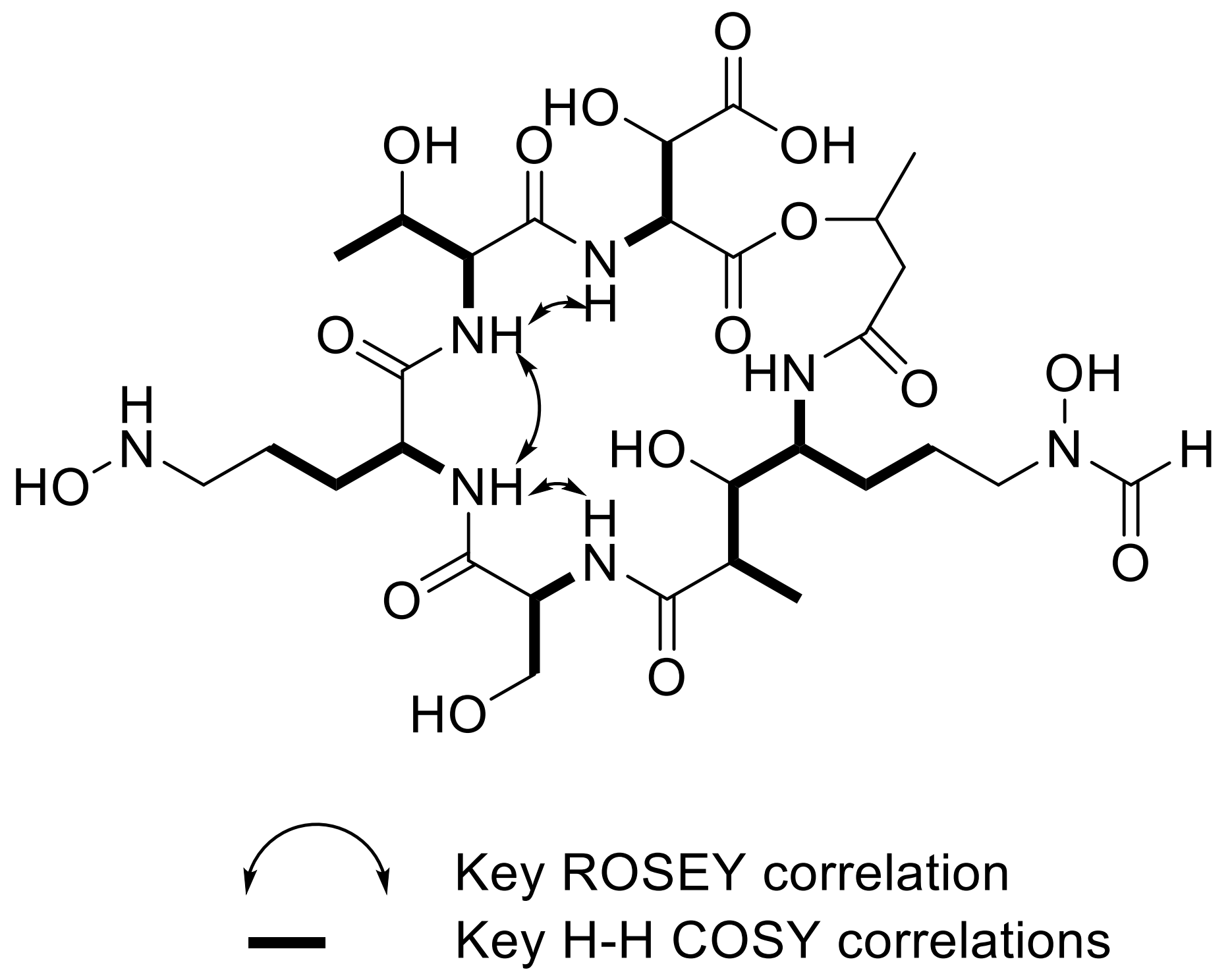

Figure S15. Key ROSEY- and COSY-NMR correlations used for the structural elucidation of VacA from MST127.

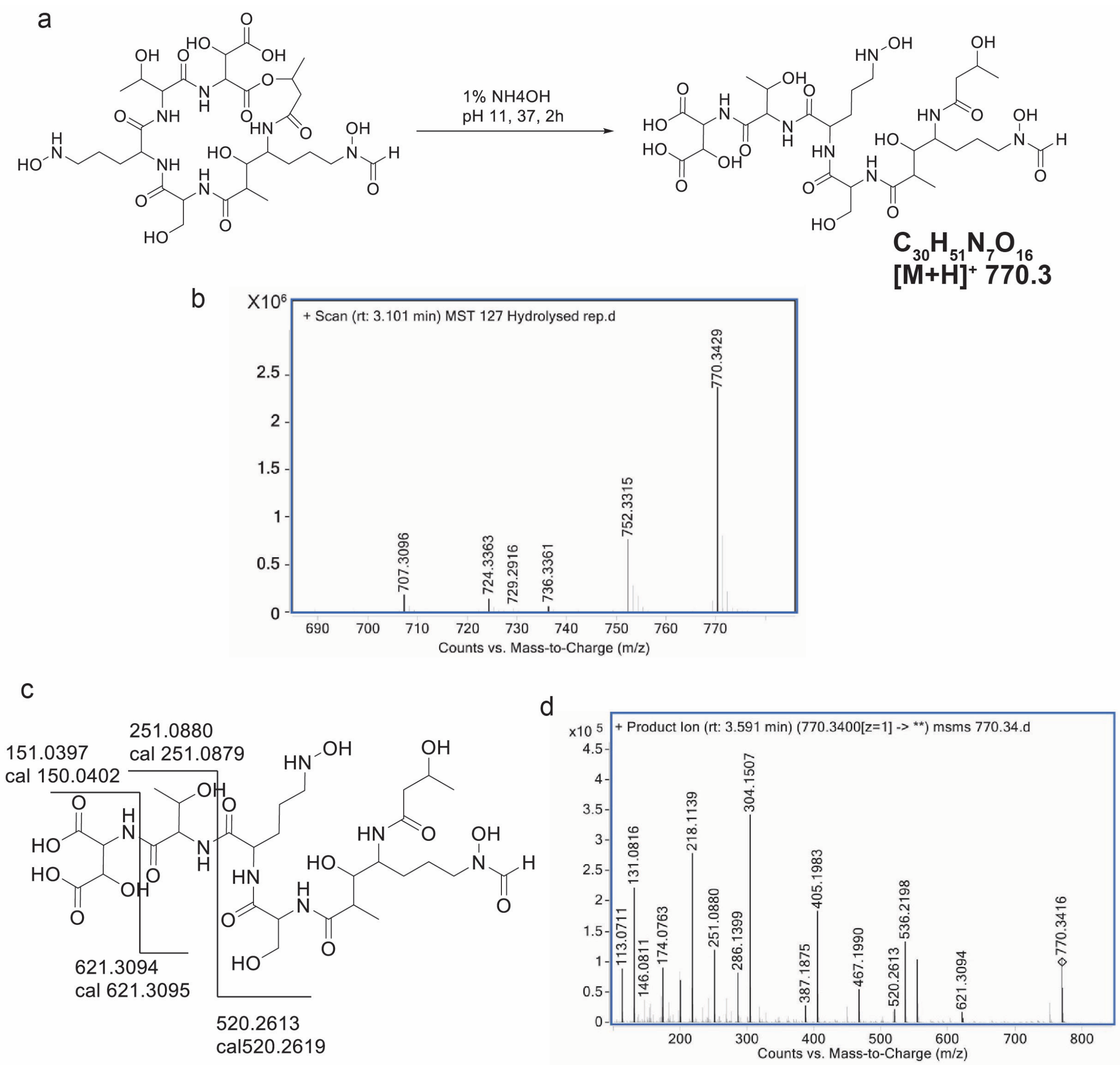

Figure S16. (a) Hydrolysis of VacA leading to ring opening. (b) Confirmation of the hydrolyzed product by mass shift: addition of 18 Da to the parent ion ($[M+H]^+$ 752.33) yields $[M+H]^+$ 770.34. (c) MS/MS fragmentation pattern and structural interpretation of intact VacA. (d) MS/MS fragmentation spectrum of hydrolyzed VacA, confirming the proposed fragmentation pattern.

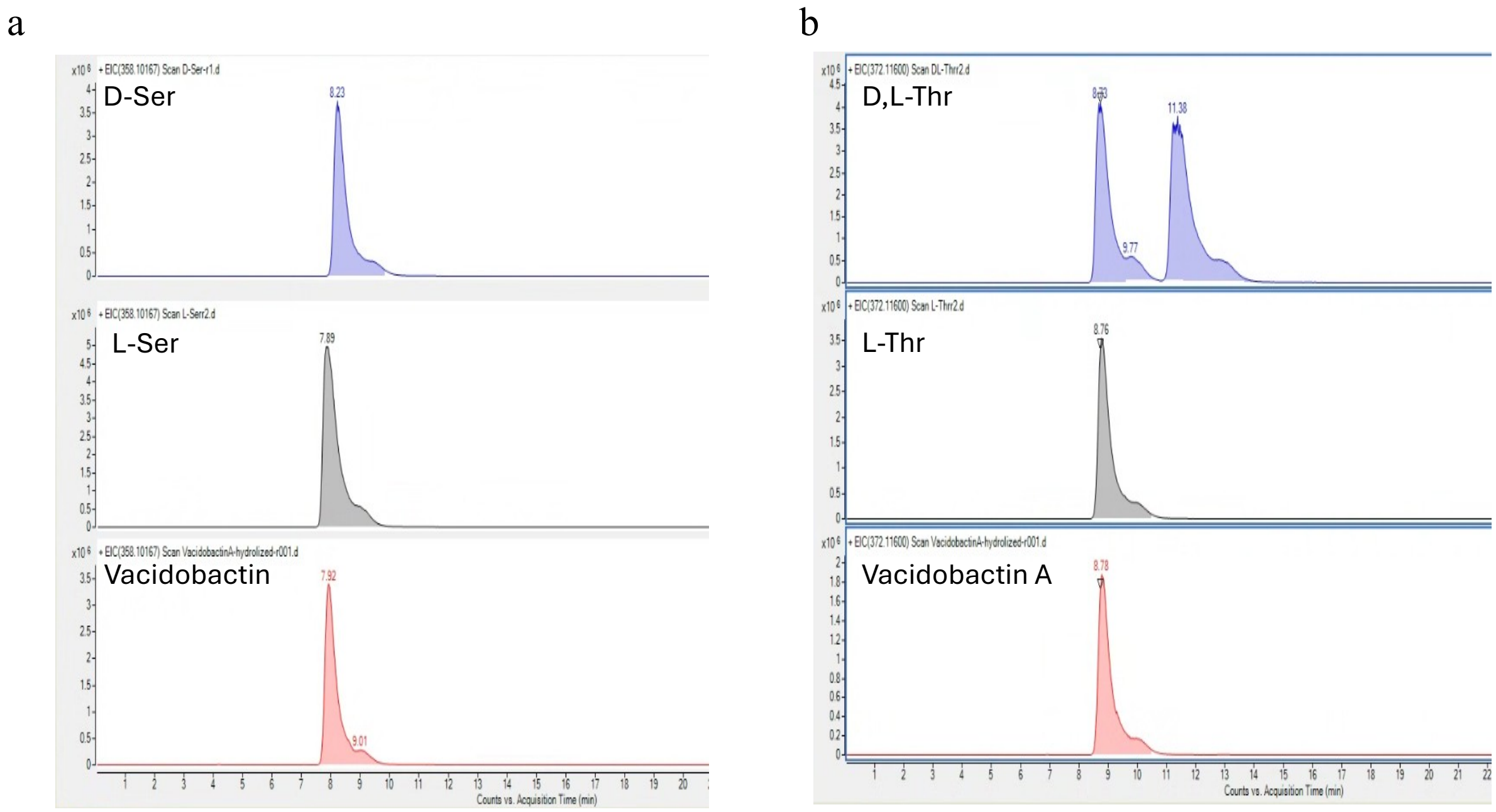

Figure S17. a) Extracted ion Chromatograms (EIC) at 358.10167, corresponding to derivatized serine; b). EIC at 372.11690, corresponding to the modified threonine. The other 3 non-proteinogenic amino acids (4-amino-3-hydroxy-7-(N-hydroxyformamido)-2-methylheptanoic acid, 2-amino-5-(hydroxyamino)pentanoic acid, and β-hydroxyaspartic acid) could not be detected using this approach.

a

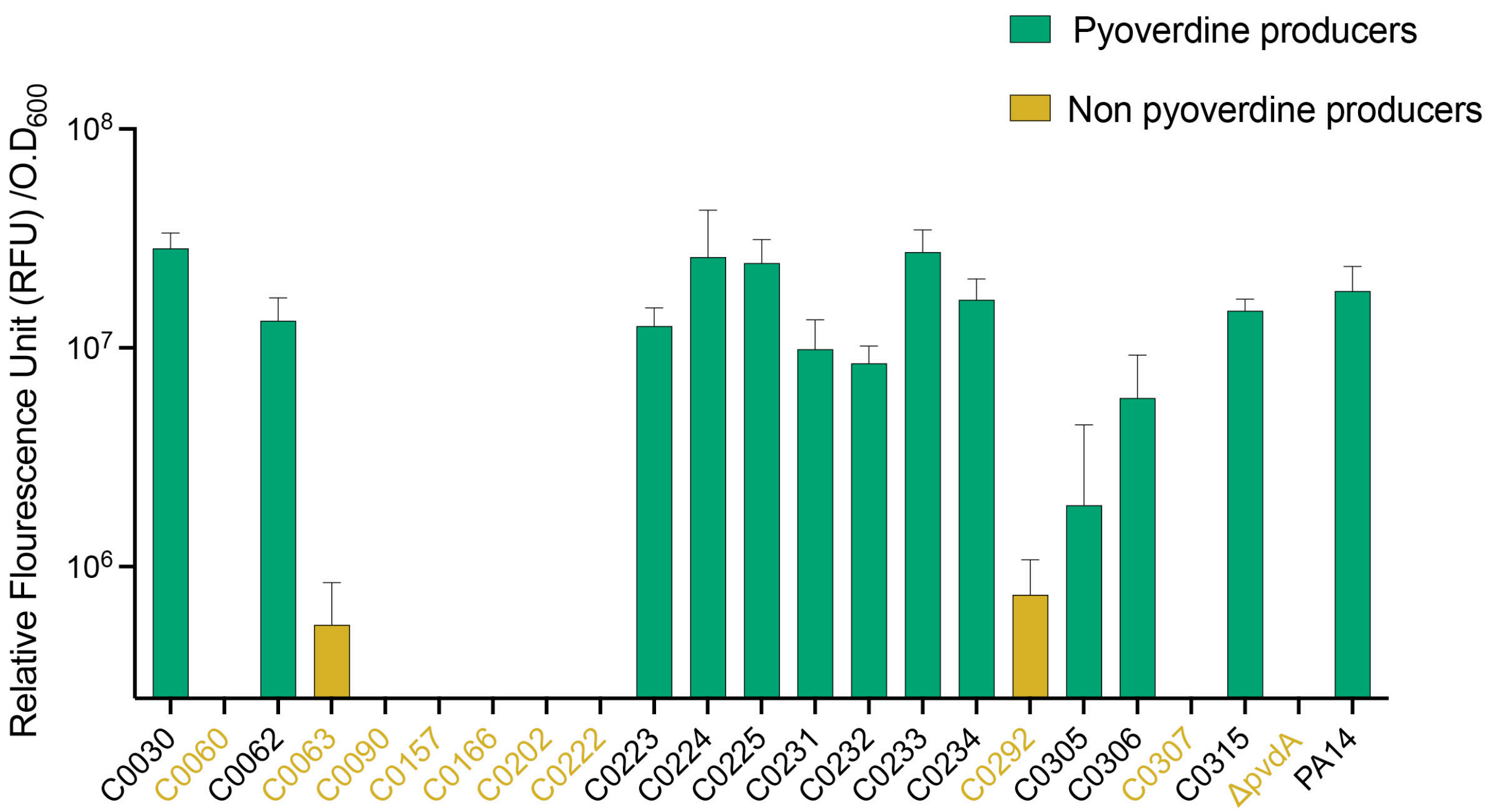

b

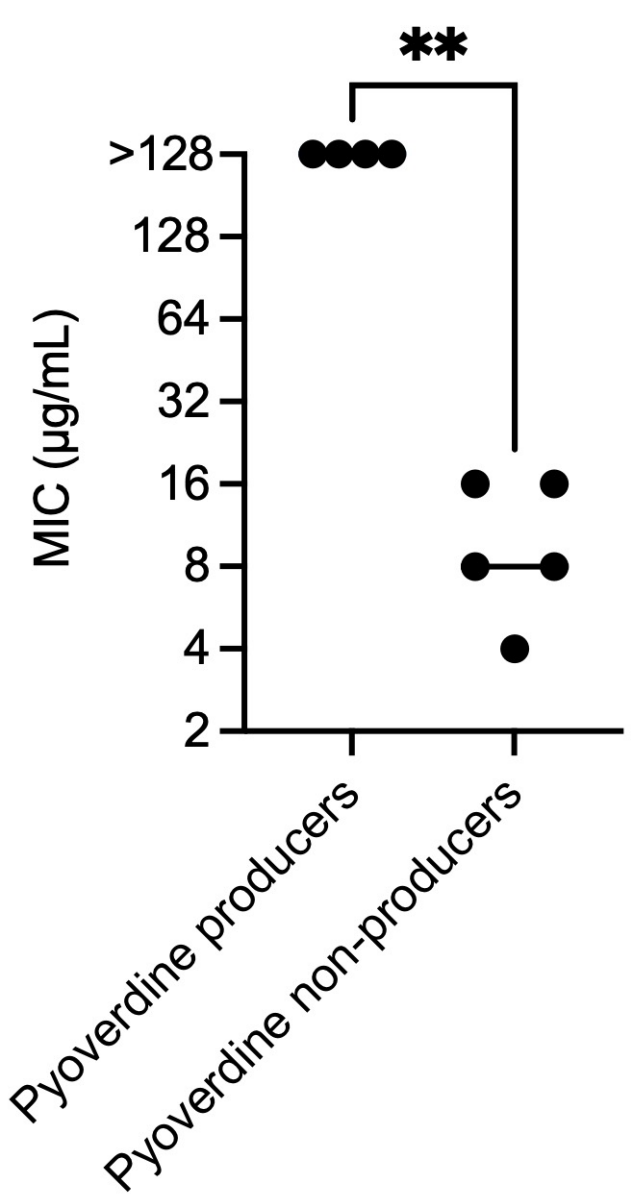

Figure S18. (a) Pyoverdine production among *P. aeruginosa* strains from the Wright clinical collection. Pyoverdine producers are indicated in green, and non-producers in yellow. The assay was performed in triplicate with three independent biological replicates. Optical density at 600 nm (OD_{600}) was consistent across all isolates, indicating that differences in fluorescence reflect true variation in siderophore production rather than growth differences. (b) Minimum Inhibitory

Concentrations (MICs) of VacA in pyoverdine-producing and non-producing *P. aeruginosa* strains. Scatter plot comparing VacA MICs (µg/mL) in pyoverdine producers (n = 4–5) versus non-producers (n = 4–5), with horizontal lines indicating median values; statistical significance determined by Mann-Whitney U test (*$p < 0.05$).

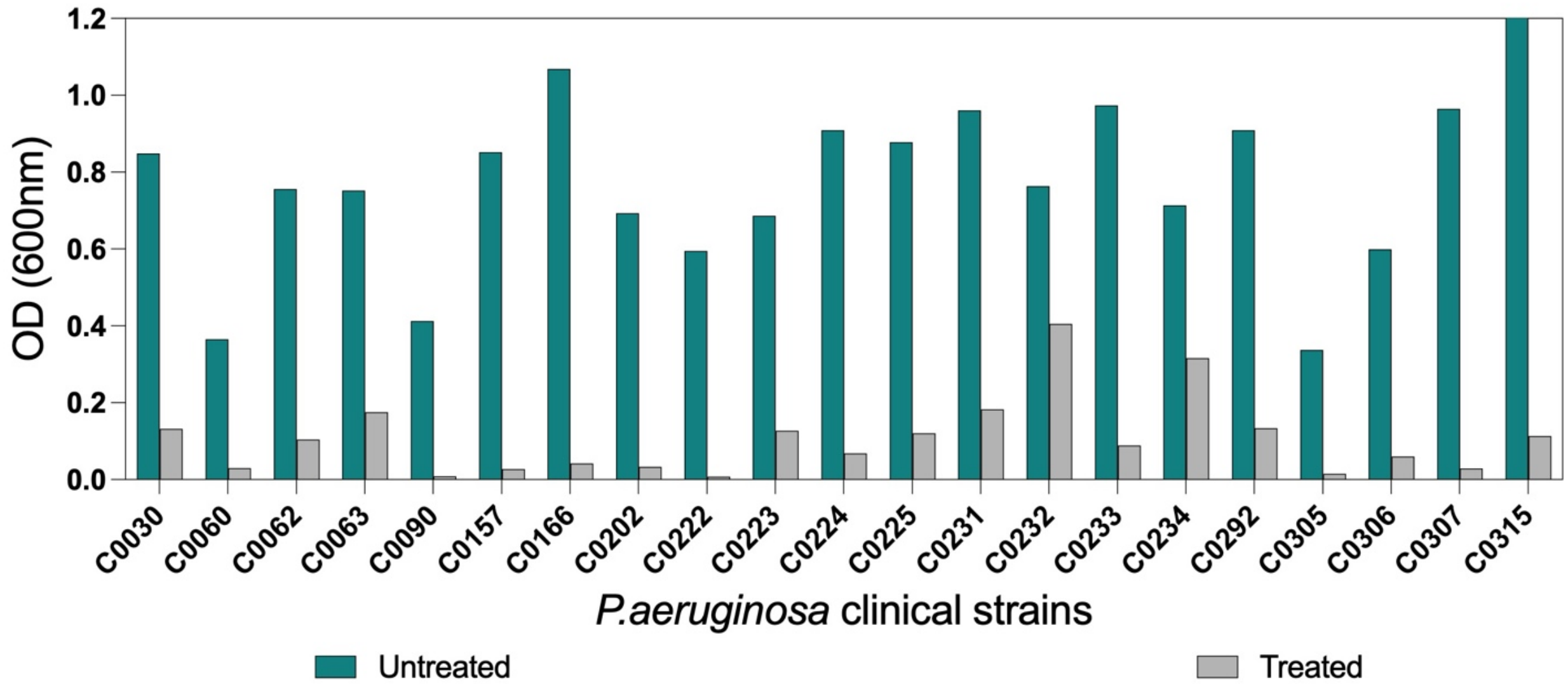

Figure S19. Bar graph representing the combination assay results against various clinical isolates of *P. aeruginosa*. The combination of VacA (8 µg/mL) and thiostrepton (0.5 µg/mL) was used in comparison to the untreated cells. The data represents mean of two replicates and representative of two biological repeats.

SUPPLEMENTARY TABLES:

Table S1. Antibiogram of *P. aeruginosa* used in this study

Strain number	Resistance profile
C0292	ampicillin, amoxicillin clavulanic acid, cefazolin, cefalotin, ciprofloxacin, cefixime, ceftazidime, metropenem, nitrofuratoin, piperacillin tazobactam, tetracycline, trimethoprim sulfamethoxazole, cefoxitin, ceftriaxone
C0030	ampicillin, amoxicillin clavulanic acid, cefazolin, cefalotin, cefixime, nitrofuratoin, tetracycline, trimethoprim sulfamethoxazole, cefoxitin, ceftriaxone
C0060	ampicillin, amoxicillin clavulanic acid, cefazolin, cefalotin, ciprofloxacin, cefixime, gentamicin, nitrofuratoin, tetracycline, trimethoprim sulfamethoxazole, cefoxitin, ceftriaxone
C0062	ampicillin, amoxicillin clavulanic acid, cefazolin, cefalotin, cefixime, nitrofuratoin, tetracycline, trimethoprim sulfamethoxazole, cefoxitin, ceftriaxone
C0063	ampicillin, amoxicillin clavulanic acid, cefazolin, cefalotin, cefixime, ceftazidime, metropenem, nitrofuratoin, piperacillin tazobactam, tetracycline, trimethoprim, sulfamethoxazole, cefoxitin, ceftriaxone
C0090	ampicillin, amoxicillin clavulanic acid, cefazolin, cefalotin, cefixime, ceftazidime, nitrofuratoin, piperacillin tazobactam, tetracycline, trimethoprim sulfamethoxazole, cefoxitin, ceftriaxone
C0157	ampicillin, amoxicillin clavulanic acid, cefazolin, cefalotin, cefixime, nitrofuratoin, tetracycline, trimethoprim sulfamethoxazole, cefoxitin, ceftriaxone
C0166	ampicillin, amoxicillin clavulanic acid, cefazolin, cefalotin, cefixime, nitrofuratoin, tetracycline, trimethoprim sulfamethoxazole, cefoxitin, ceftriaxone
C0202	ampicillin, amoxicillin clavulanic acid, cefazolin, cefalotin, cefixime, nitrofuratoin, tetracycline, trimethoprim sulfamethoxazole, cefoxitin, ceftriaxone
C0222	ampicillin, amoxicillin clavulanic acid, cefazolin, cefalotin, ciprofloxacin, cefixime, nitrofuratoin, tetracycline, trimethoprim sulfamethoxazole, cefoxitin, ceftriaxone
C0223	ampicillin, amoxicillin clavulanic acid, cefazolin, cefalotin, ciprofloxacin, cefixime, nitrofuratoin, tetracycline, trimethoprim sulfamethoxazole, cefoxitin, ceftriaxone
C0224	ampicillin, amoxicillin clavulanic acid, cefazolin, cefalotin, ciprofloxacin, cefixime, nitrofuratoin, tetracycline, trimethoprim sulfamethoxazole, cefoxitin, ceftriaxone
C0225	ampicillin, amoxicillin clavulanic acid, cefazolin, cefalotin, cefixime, nitrofuratoin, tetracycline, trimethoprim sulfamethoxazole, cefoxitin, ceftriaxone

C0231	ampicillin, amoxicillin clavulanic acid, cefazolin, cefalotin, cefixime, nitrofuratoin, tetracycline, trimethoprim sulfamethoxazole, cefoxitin, ceftriaxone
C0232	ampicillin, amoxicillin clavulanic acid, cefazolin, cefalotin, cefixime, nitrofuratoin, tetracycline, trimethoprim sulfamethoxazole, cefoxitin, ceftriaxone
C0233	ampicillin, amoxicillin clavulanic acid, cefazolin, cefalotin, ciprofloxacin, cefixime, nitrofuratoin, tetracycline, trimethoprim sulfamethoxazole, cefoxitin, ceftriaxone
C0234	ampicillin, amoxicillin clavulanic acid, cefazolin, cefalotin, cefixime, nitrofuratoin, tetracycline, trimethoprim sulfamethoxazole, cefoxitin, ceftriaxone
C0305	ampicillin, amoxicillin clavulanic acid, cefazolin, cefalotin, cefixime, nitrofuratoin, tetracycline, trimethoprim sulfamethoxazole, cefoxitin, ceftriaxone
C0306	ampicillin, amoxicillin clavulanic acid, cefazolin, cefalotin, cefixime, nitrofuratoin, tetracycline, trimethoprim sulfamethoxazole, cefoxitin, ceftriaxone
C0307	ampicillin, amoxicillin clavulanic acid, cefazolin, cefalotin, cefixime, nitrofuratoin, tetracycline, trimethoprim sulfamethoxazole, cefoxitin, ceftriaxone
C0315	ampicillin, amoxicillin clavulanic acid, cefazolin, cefalotin, cefixime, nitrofuratoin, piperacillin tazobactam, tetracycline, trimethoprim sulfamethoxazole, cefoxitin,<br>ceftriaxone

Table S2. Strains used in this study

	Source
Variovorax paradoxus (MST127)	This study
P. aeruginosa (C0292)	This study
K. pneumoniae (C0026)	This study
A. baumanii complex (C0286)	This study
S. aureus (C0653)	This study
A. baumanii (ATCC 17978)	This study
S. aureus (ATCC 29213)	This study
K. pneumoniae (C0298)	This study
K. pneumoniae (ATCC 33495)	This study
P. aeruginosa (PA01)	This study
P. aeruginosa (381)	This study
P. aeruginosa (255)	This study
P. aeruginosa (C0060)	This study
P. aeruginosa (C0030)	This study
P. aeruginosa (C0090)	This study
P. aeruginosa (C0166)	This study
P. aeruginosa (C0307)	This study
P. aeruginosa (C0056)	This study
P. aeruginosa (C0029)	This study
P. aeruginosa (C0139)	This study
P. aeruginosa (167a) NG	This study
P. aeruginosa (167c) NG	This study
P. aeruginosa (C0063)	This study
P. aeruginosa (C0222)	This study
P. aeruginosa (C0202)	This study
P. aeruginosa (C0157)	This study
P. aeruginosa (C0139)	This study
PA14 *fpvA::*Mar2xT7 PA2398 Type 1 ferripyoverdine receptor	(1)
PA14 *fpvB::*Mar2xT7 PA4168 Alternate Type 1 ferripyoverdine receptor	(1)
PA14 *ΔfpvA fpvB::Mar2xT7* ,	(1)
PA14 *foxA::*Mar2xT7 PA2466 Ferrioxamine receptor FoxA	(1)
PA14 *fiuA::*Mar2xT7 PA0470 Ferrichrome receptor FiuA	(1)
PA14 tonB1::Mar2xT7	(1)
E. coli DH5α	Invitrogen
E. coli DH5α pEX18Gm-Δ*pvdA*	This study
E. coli DH5α pEX18Gm-Δ*pchA*	This study
E. coli DH5α pHERD20T-*fiuA*	This study
E. coli DH5α pHERD20T-N_{fiuA}-MST127	This study
E. coli SM10	Invitrogen

E. coli SM10 pEX18Gm-Δ*pvdA*	This study
E. coli SM10 pEX18Gm-Δ*pchA*	This study
P. aeruginosa PA14	(1)
P. aeruginosa PA14 Δ*pvdA* Δ*pchA*	(4)
P. aeruginosa PA14 Δ*pvdA* Δ*pchA* pHERD20T	This study
P. aeruginosa PA14 Δ*pvdA* Δ*pchA* pHERD20T-*fiuA*	This study
P. aeruginosa PA14 Δ*pvdA* Δ*pchA* pHERD20T-N_{fiuA}-MST127	This study
Variovorax Paradoxus DSM30334	(12)

Table S3. Plasmids used in this study

	Source
pEX18Gm	(2)
pEX18Gm-Δ*pvdA*	This study
pEX18Gm-Δ*pchA*	This study
pHERD20T	(3)
pHERD20T-*fiuA*	This study
pHERD20T-N_{fiuA}-MST127	This study

Table S4. Primers used in this study. Restriction enzyme sites are bolded and underlined. Overlapping regions are italicized.

Primer	5' → 3'
Δ*pvdA* upstream FWD	GCC**GAATTC**CATTATCGGGAGCTGCTGCGATTC
Δ*pvdA* upstream REV	CAAT**GGATCC**TTCCAGTTCCTCTGGATTGGCCCC
Δ*pvdA* downstream FWD	CAAT**GGATCC**ACGCAGGGCGTTTTCGTTGCATGT
Δ*pvdA* downstream REV	CAAT**AAGCTT**GAAGTGGCTGGAGGTATCCACCGT
Δ*pchdA* upstream FWD	CAAT**GAATTC**TTCCGTAGCTGGAGCGAACGCCTG
Δ*pchdA* upstream REV	TCGACGACGACATTCCGTTCTAA*TCATGCGGCACCCCGTGTCTGGCG*
Δ*pchA* downstream FWD	*CGCCAGACACGGGGTGCCGCATGA*TTAGAACGGAATGTCGTCGTCGA
Δ*pchA* downstream REV	CAAT**AAGCTT**GTGACCAGCATTCGCCTGCCGCTC
N-*fiuA* FWD	CAAT**GAATTC**AGACTCGTTTCCATCCAGGAGC
N-*fiuA* REV	TCGGTCTTGGTGCCGGTG*GCGCTGCGCTTGGCCACGTA*
V. paradoxus TBDT FWD	*TACGTGGCCAAGCGCAGC*GCCACCGGCACCAAGACCGA
V. paradoxus TBDT REV	CAAT**AAGCTT**GCCTTTGGAGGCCTGCCG

Table S5. H and C NMR Data of compound VacA from MST127

No.	δ_H	δ_C	No.	δ_H	δ_C
1	1.20 (d, J = 6.3 Hz)	20.1	19		171.0
2	5.21 (m)	69.2	20 NH	8.26 (br s)	
3	2.42 (br d, J = 15.1 Hz)<br>2.76 (dd, J = 15.1, 8.4 Hz)	39.1	21	4.13 (m)	54.0
4		169.7	22	1.67 (m), 1.48 (m)	29.1
5 NH	7.83 (br s)		23	1.66 (m), 1.34 (m)	21.6
6	3.19 (m)	52.0	24	3.81 (m), 3.02 (m)	47.4
7	1.56 (m)	22.5	26		172.0
8	1.77(m), 1.59 (m)	22.4	27 NH	7.65 (br s)	
9	3.52 (m), 3.44(m)	45.4	28	3.96 (dd, J = 4.4, 8.0 Hz)	60.6
11	8.28 (s)	161.9	29	4.08 (m)	65.5
12	3.75 (d, J = 9.3 Hz)	72.5	30	1.03 (d, J = 6.2 Hz)	20.3
13	2.57 (m)	42.6	31		170.2
14	0.94 (d, J = 6.8 Hz)		32 NH	7.22 (d, J = 6.8 Hz)	
15		174.5	33	4.77 (br d, J = 6.8 Hz)	55.3
16 NH	8.16 (d, J = 6.6 Hz)		34	4.22 (d, J = 2.4 Hz)	70.6
17	4.30 (m)	56.1	35		172.3
18	3.70(m), 3.65(m)	61.4	36		167.7

Table S6. The comparison of between the genes and their putative functions from BGCs

**Vacidobactin gene cluster analysis (*Variovorax paradoxus* S110 (DSM No. 30034)**				**Vacidobactin gene cluster analysis (Variovorax paradoxus MST 127)**				
**Locus**	**Predicted Function**	**Strand**	**Amino Acids**	**Locus**	**Location**	**Predicted Function**	**Strand**	**Amino acids**
Vapar_3752	RNA polymerase, sigma-24 subunit, ECF subfamily	-	181	ctg8_230	248,867 - 249,412	Probable RNA polymerase sigma factor FecI	-	181
Vapar_3751	anti-FecI sigma factor	-	67	ctg8_229	248,567 - 248,770	no-hits	-	67
Vapar_3750	MbtH domain protein	-	85	ctg8_228	248,209 - 248,466	mbtH-like protein	-	85
Vapar_3749	Thioesterase	-	246	ctg8_227	247,466 - 248,203	thioesterase	-	245
Vapar_3748	Phosphopantetheinyl transferase	-	229	ctg8_226	246,799 - 247,488	4'-phosphopantetheinyl transferase	-	229
Vapar_3747	TauD dioxygenase	-	330	ctg8_225	245,810 - 246,802	Dioxygenase TauD/TfdA	-	330
Vapar_3746	NRPS	-	1776	ctg8_224	240,474 - 245,789	NRPS	-	1771
Vapar_3745	PKS	-	1520	ctg8_223	235,912 - 240,474	PKS	-	1520
Vapar_3744	NRPS	-	1110	ctg8_222	232,574 - 235,915	NRPS	-	1113
Vapar_3743	NRPS	-	2626	ctg8_221	224,697 - 232,574	NRPS	-	2625
Vapar_3742	NRPS	-	1358	ctg8_220	220,627 - 224,700,	NRPS	-	1357
Vapar_3741	TonB siderophore receptor	+	723	ctg8_219	218,377 - 220,545	TonB-dependent Receptor	+	722
Vapar_3740	L-lysine 6-monooxygenase	+	439	ctg8_218	216,862 - 218,214,	lysine/ornithine N-monooxygenase	+	450
Vapar_3739	N-acetyltransferase	+	344	ctg8_217	215,770 - 216,810	Acetyltransferase (GNAT)	+	346
Vapar_3738	N5-hydroxyornithine formyltransferase	+	281	ctg8_216	214,928 - 215,773	hydroxymethyl-, formyl- and related transferase activity	+	302

Vapar_3737	Ferric iron reductase	+	281	ctg8_215	214,047 - 214,826	Ferric iron reductase FhuF-like transporter	+	259
Vapar_3736	Cyclic peptide transporter	-	563	ctg8_214	212,151 - 213,842	ABC_membrane, ABC_transporter	-	563

References-Supplementary

1. NT L, JM U, S M, et al. An ordered, nonredundant library of Pseudomonas aeruginosa strain PA14 transposon insertion mutants. *Proceedings of the National Academy of Sciences of the United States of America*. 2006;103(8):2833-2838. doi:10.1073/PNAS.0511100103
2. TT H, RR KS, AJ K, HP S. A broad-host-range Flp-FRT recombination system for site-specific excision of chromosomally located DNA sequences: application for isolation of unmarked Pseudomonas aeruginosa mutants. *Gene*. 1998;212(1):77-86. doi:10.1016/S0378-1119(98)00130-9
3. Qiu D, Damron FH, Mima T, Schweizer HP, Yu HD. PBAD-Based Shuttle Vectors for Functional Analysis of Toxic and Highly Regulated Genes in Pseudomonas and Burkholderia spp. and Other Bacteria. *Applied and Environmental Microbiology*. 2008;74(23):7422. doi:10.1128/AEM.01369-08
4. Chan DCK, Burrows LL. Pseudomonas aeruginosa FpvB Is a High-Affinity Transporter for Xenosiderophores Ferrichrome and Ferrioxamine B. *mBio*. 2022;0(0):e03149-22. doi:10.1128/mbio.03149-22
